# Supplementary material for: Self-organizing layers from complex molecular anions
Source: Nat Commun. 2018 May 14;9:1889. doi: 10.1038/s41467-018-04228-2 (PMC5951818; doi:10.1038/s41467-018-04228-2)
Supplement: Supplementary file 1 — Supplementary Information [file 41467_2018_4228_MOESM1_ESM.pdf]

Supplementary Information

to

Self-organizing layers from complex molecular anions

Warneke et al.

Jonas Warneke[1], Martin E. McBriarty[1], Shawn L. Riechers[1], Swarup China[2], Mark H. Engelhard[2], Edoardo Aprà[2], Robert P. Young[2], Nancy M. Washton[2], Carsten Jenne[3], Grant E. Johnson[1], Julia Laskin[1,4]

1) Physical Sciences Division, Pacific Northwest National Laboratory, 902 Battelle Boulevard, P.O. Box 999, MSIN K8-88, Richland WA, 99352, USA

2) Environmental Molecular Sciences Laboratory, Pacific Northwest National Laboratory, P. O. Box 999, Richland, WA 99352, USA

3) Fachbereich C - Anorganische Chemie, Bergische Universität Wuppertal, Gaußstraße 20, 2119 Wuppertal, Germany

4) Department of Chemistry Purdue University West Lafayette IN 47907 USA

Table of content

Supplementary Figures2

Color code: Data-Method assignment

Optical microscopy (opt), Atomic force microscopy (AFM), Infrared Reflection-Absorption Spectroscopy (IR), mass spectrometry analysis (MS), X-ray photoelectron spectroscopy (XPS), Nuclear Magnetic Resonance (NMR), computational investigations (Comp.)

Supplementary Figures	Data	Page
Supplementary Figure 1: Capacitor		4
Supplementary Figure 2: Bright field image deposition spot prior to dewetting	(opt)	5
Supplementary Figure 3: Comparison roughness of layer and substrate	(AFM)	6
Supplementary Figure 4: Comparison hole borders at different layer thickness	(AFM)	7
Supplementary Figure 5: Hole border development and merging of two borders	(AFM)	7
Supplementary Figure 6: Thickness dependency of self-organized structure	(opt)	8
Supplementary Figure 7: Hydrocarbon accumulation proportional to ion signal	(IR)	9
Supplementary Figure 8: $[B_{12}F_{12}]^{2-/1-/0}$ vibrational frequencies	(IR),(Comp.)	10
Supplementary Figure 9: $[B_{12}Cl_{12}]^{2-/1-/0}$ vibrational frequencies	(IR),(Comp.)	10
Supplementary Figure 10: IR spectra dependent on halogen ligand X in anion	(IR)	11
Supplementary Figure 11: MS/MS of phthalates	(MS)	13
Supplementary Figure 12: MS/MS of phthalates	(MS)	14
Supplementary Figure 13: XPS spectrum	(XPS)	15
Supplementary Figure 14: Fitting of the carbon XPS data	(XPS)	16
Supplementary Figure 15: NMR chemical shift ^{11}B	(NMR)	17
Supplementary Figure 16: ^{1}H NMR	(NMR)	18
Supplementary Figure 17: Comparison dewetting of $[B_{12}F_{12}]^{2-}$ and $[B_{12}Cl_{12}]^{2-}$ layers	(opt)	19
Supplementary Figure 18: Comparison droplets $[B_{12}F_{12}]^{2-}$ and $[B_{12}Cl_{12}]^{2-}$	(AFM)	20
Supplementary Figure 19: Comparison line profiles of droplets (X=F,Cl,Br,I)	(AFM)	20
Supplementary Figure 20: Layer appearance after vacuum drying	(opt)	21
Supplementary Figure 21: Development $[B_{12}I_{12}]^{2-}$ based layer over weeks	(opt)	22
Supplementary Figure 22: Water induced dewetting of a $[B_{12}Br_{12}]^{2-}$ based layer	(opt)	22
Supplementary Figure 23,24: Comparison dewetting on FSAM and HSAM	(opt)	23
Supplementary Figure 25: Changing phthalate composition	(MS), (opt)	24
Supplementary Figure 26: RGA spectrum during defined phthalate experiment	(MS)	25
Supplementary Figure 26: Defined phthalate experiment (Layer analysis)	(MS), (opt)	26
Supplementary Figure 28: Glycine experiment (partial substitution)	(opt), (IR)	27
Supplementary Figure 29: Detailed analysis tips prior to and after washing.	(AFM)	28
Supplementary Figure 30: Optimized geometry $[B_{12}Cl_{12}]^{2-}$ + H_2O	(Comp.)	29
Supplementary Figure 31: Optimized geometry $[B_{12}Cl_{12}]^{2-}$ + diisodecylphthalate	(Comp.)	29
Supplementary Figure 31: Optimized geometry $[B_{12}Cl_{12}]^{2-}$ + 2*diisodecylphthalate	(Comp.)	29
Supplementary Figure 33: Contact angles phthalates on different surfaces	(opt)	30
Supplementary Figure 34: Scheme water binding to B12F12 and B12I12.	(Comp.)	31
Supplementary Figure 35: Salts on surface.	(opt)	33

Supplementary Tables

Supplementary Table 1: Molecular formulas Hydrocarbons (MS) 12
Supplementary Table 2: XPS elemental composition after dewetting (XPS) 16
Supplementary Table 3: Optimized geometry $[B_{12}F_{12}]^{2-}$ + H_2O (Comp.) 31
Supplementary Table 4: Optimized geometry $[B_{12}I_{12}]^{2-}$ + H_2O (Comp.) 32

Supplementary Notes

Supplementary Note 1: Parallel plate capacitor model for ion soft landing 4
Supplementary Note 2: Bright field image deposition spot before dewetting 5
Supplementary Note 3: AFM different stage of dewetting ($[B_{12}Cl_{12}]^{2-}$ based layer) 6
Supplementary Note 4: Thickness dependency of self-organized structure 8
Supplementary Note 5: IR investigations 9
Supplementary Note 6: Mass spectrometry analysis of layer 12
Supplementary Note 7: XPS analysis 14
Supplementary Note 8: NMR analysis 17
Supplementary Note 9: XRD analysis 18
Supplementary Note 10: Morphology of the $[B_{12}F_{12}]^{2-}$ layer and droplets 19
Supplementary Note 11: Vacuum drying 21
Supplementary Note 12: Dewetting of $[B_{12}Br_{12}]^{2-}$ and $[B_{12}I_{12}]^{2-}$ based layer 22
Supplementary Note 13: Layers on HSAM 23
Supplementary Note 14: Phthalate composition 24
Supplementary Note 15: Codeposition of a defined phthalate 25
Supplementary Note 16: Glycine codeposition experiment 27
Supplementary Note 17: Details tips analysis before and after washing 28
Supplementary Note 18: Bindung of diisodecylphthalate and water to $[B_{12}Cl_{12}]^{2-}$ 29
Supplementary Note 19: Correlation phthalate contact angle and final morphology on surfaces 30
Supplementary Note 20: Binding of water to $[B_{12}F_{12}]^{2-}$ and $[B_{12}I_{12}]^{2-}$ in comparison. 31
Supplementary Note 21: Dropcasted $[B_{12}X_{12}]^{2-}$ salt solutions. 33

Supplementary Note 1: Parallel plate capacitor model for ion soft landing

A scheme to rationalize the retention of charge during ion soft landing is shown in Supplementary Figure 1. Note that deposition of cations on bare metal surfaces results in neutralization. In contrast, deposition on an insulating surface results in buildup of electric fields that prevent further ion deposition at high coverages. Larger amounts of ions that retain their charges can be deposited onto insulating SAMs on top of an underlying conductive surface. The landing of a cation on such a substrate induces a mirror charge in the gold that is grounded through a picoammeter. The situation can be described as loading a two-plate capacitor. If the layer of soft landed ions and mirror charges is approximated by uniformly charged round plates with radius R separated by a distance d (Supplementary Figure 1c), the force F on an ion approaching the center of the plate at distance D may be expressed using equation S1:[1]

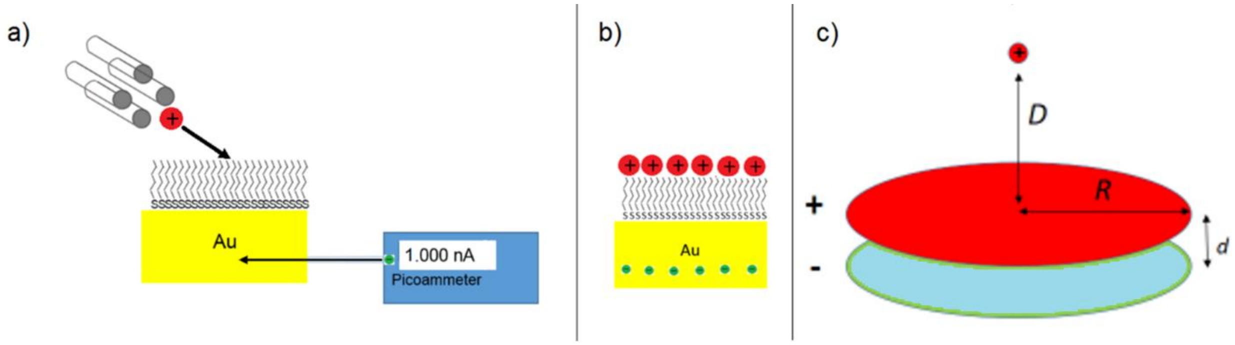

Supplementary Figure 1. (a). For every deposited ion an electron flows for charge compensation to the surface which can be measured as a current. This results in the build-up of a capacitor (b). The electric field can be approximated by Supplementary equation 1, the meaning of the variables is visualized in Figure c.

$$F = \frac{Z^2}{2\varepsilon_0} \frac{N_{\mathrm{ions}}}{A} \left(-\frac{D}{\sqrt{R^2 + D^2}} + \frac{D+d}{\sqrt{R^2 + (D+d)^2}} \right) \quad \text{(Supplementary equation 1)}$$

The electric field above the center of the plates vanishes for infinitely large plates, and, therefore, becomes extremely small for large capacitors (very large R/d ratio). However, at the borders, side effects are present that result in larger electric fields that may lead to repulsion between the deposited ions of the same polarity. Therefore, such side effects tend to be minimized by forming a smooth layer instead of many “islands” of accumulated ions (i.e. formation of one large capacitor with uniform plates vs. many small capacitors in the “island model”).

High coverages of cations usually resulted in discharge of the ions. This breakdown of the capacitor was explained by the buildup of an electric field across the insulating layer that is

strong enough to allow electrons to pass from the gold surface through the SAM.[2] The neutralization is energetically favorable since electron attachment to cations is a spontaneous process. Recent studies showed that highly electronically stable anions stay charged after deposition (see the main text). The deposition of higher amounts of anions may be possible because excess electrons can be bound to anions by several eV. In addition, electron loss from multiply charged anions is hindered by a repulsive Coulomb barrier. This may result in the build up of larger potentials. A detailed examination of the physical situation is beyond the scope of this study, but we present data that clearly show that most of the deposited ions are not neutralized (Supplementary Figure 8 and 9).

Supplementary Note 2: Bright field image of deposition spot before dewetting

The optical appearance of a deposited spot before the dewetting process starts is shown in the following image. At this stage, the layer is invisible in the dark field. For $[B_{12}Cl_{12}]^{2-}$ dewetting usually starts too soon to map the surface. Therefore, an image of a $[B_{12}Br_{12}]^{2-}$ layer is shown, which is similar in optical appearance to $[B_{12}Cl_{12}]^{2-}$ before dewetting.

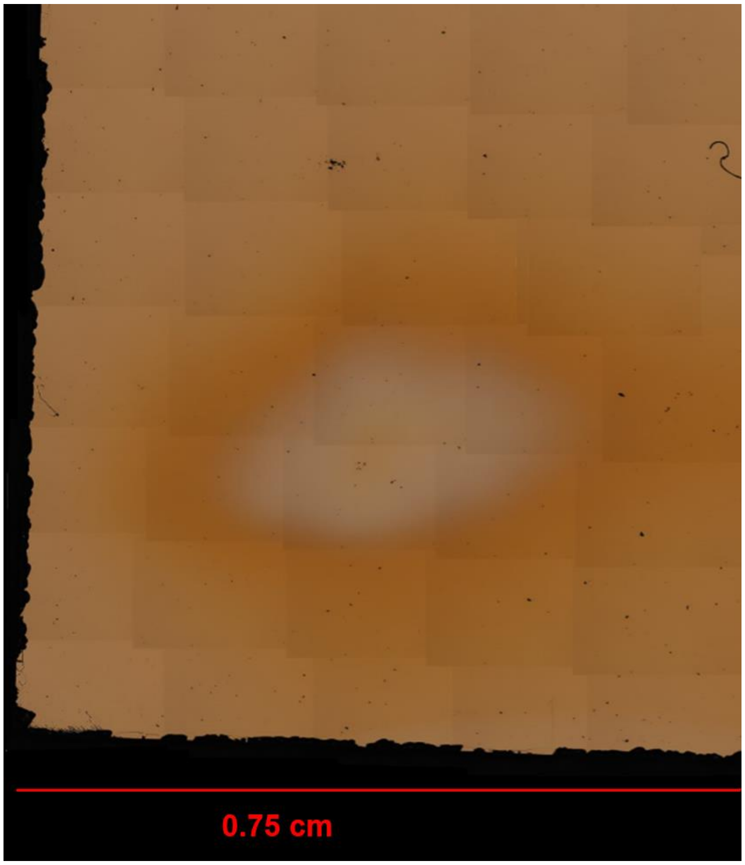

Supplementary Figure 2. Bright field optical microscope image of a layer generated by soft landing of approximately 10^{15} $[B_{12}Br_{12}]^{2-}$ ions.

Supplementary Note 3: AFM investigation of layers in the initial state of dewetting

The initially smooth morphology of the $[B_{12}Cl_{12}]^{2-}$ based layer was demonstrated by acquiring AFM images of a freshly prepared surface directly after its exposure to air. Supplementary Figure 3 demonstrates that the layer surface is much smoother than the underlying substrate (FSAM on gold coated Si). The calculated RMS roughness is 2.30 nm for the bare FSAM surface and 0.44 nm for the layer, respectively.

To estimate the thickness of the layers, we also performed AFM measurements on layers during dewetting. Supplementary Figure 4 shows line profiles obtained over the borders of growing holes. Supplementary Figure 5 shows the development in height of these borders during the process of continuous hole expansion and merging of two borders.

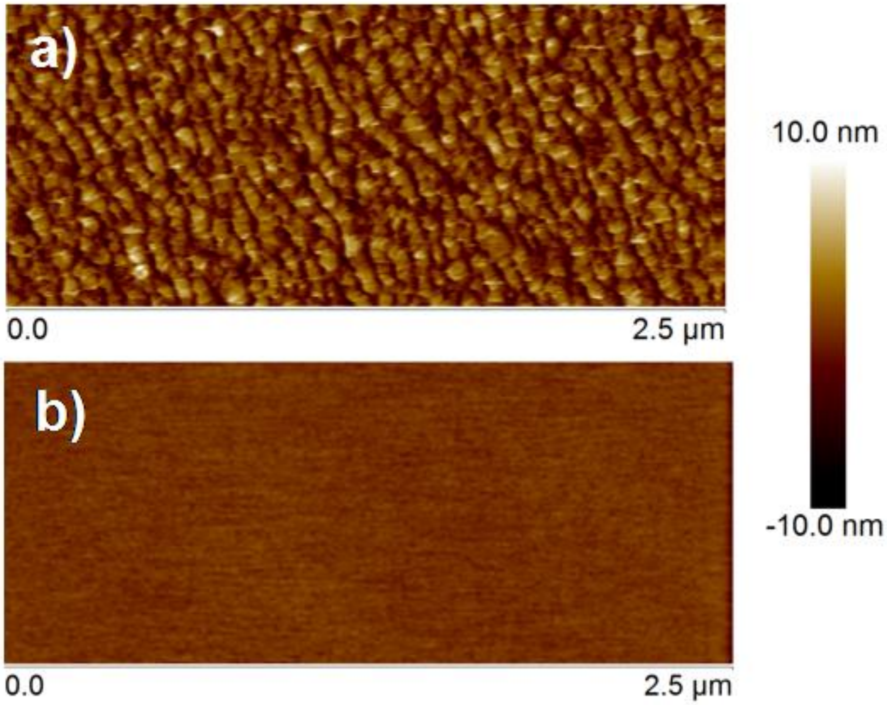

Supplementary Figure 3. AFM images showing a) a bare FSAM surface and b) the surface of a $[B_{12}Cl_{12}]^{2-}$ layer after short exposure to air before dewetting starts.

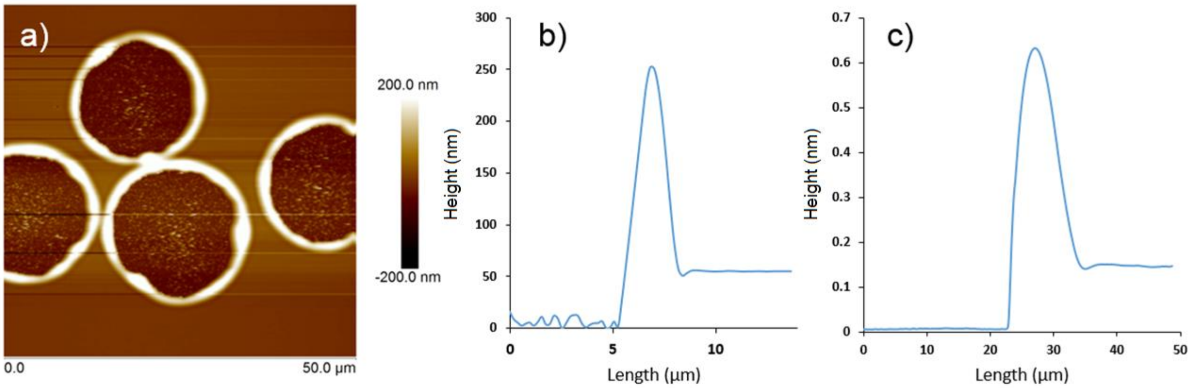

Supplementary Figure 4. a) AFM image and b) line profile over the border of a growing hole in a $[B_{12}Cl_{12}]^{2-}$ based layer after deposition of roughly $9*10^{14}$ ions. For comparison an equivalent line profile on a surface with coverage $3*10^{15}$ ions is shown in c, please note the different y-axis scale.

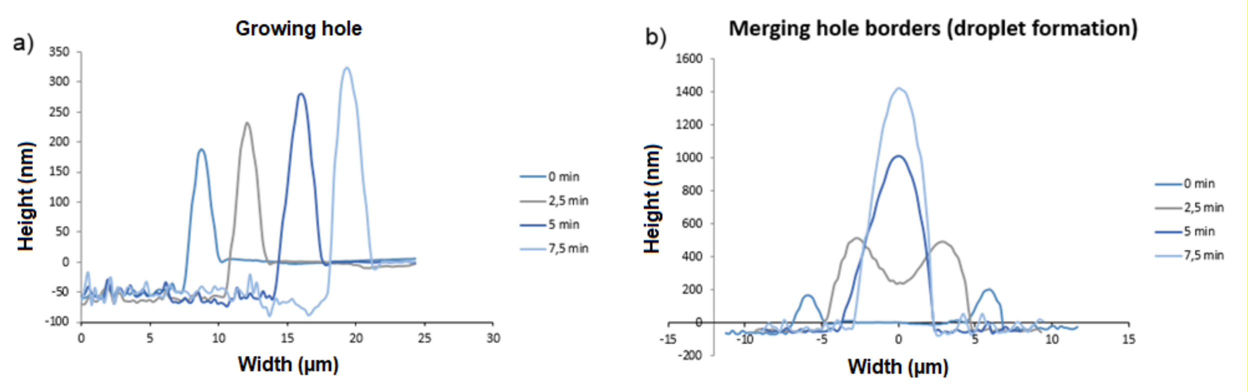

Supplementary Figure 5. Development of hole border during dewetting ($[B_{12}Cl_{12}]^{2-}$ based layer, roughly $9*10^{14}$ ions, a) a continuously growing hole, b) two merging hole borders.

Supplementary Note 4: Thickness dependency of self-organized structures

An increase in droplet size and size of the formed pattern depending on the amount of deposited $[B_{12}Cl_{12}]^{2-}$ ions was found.

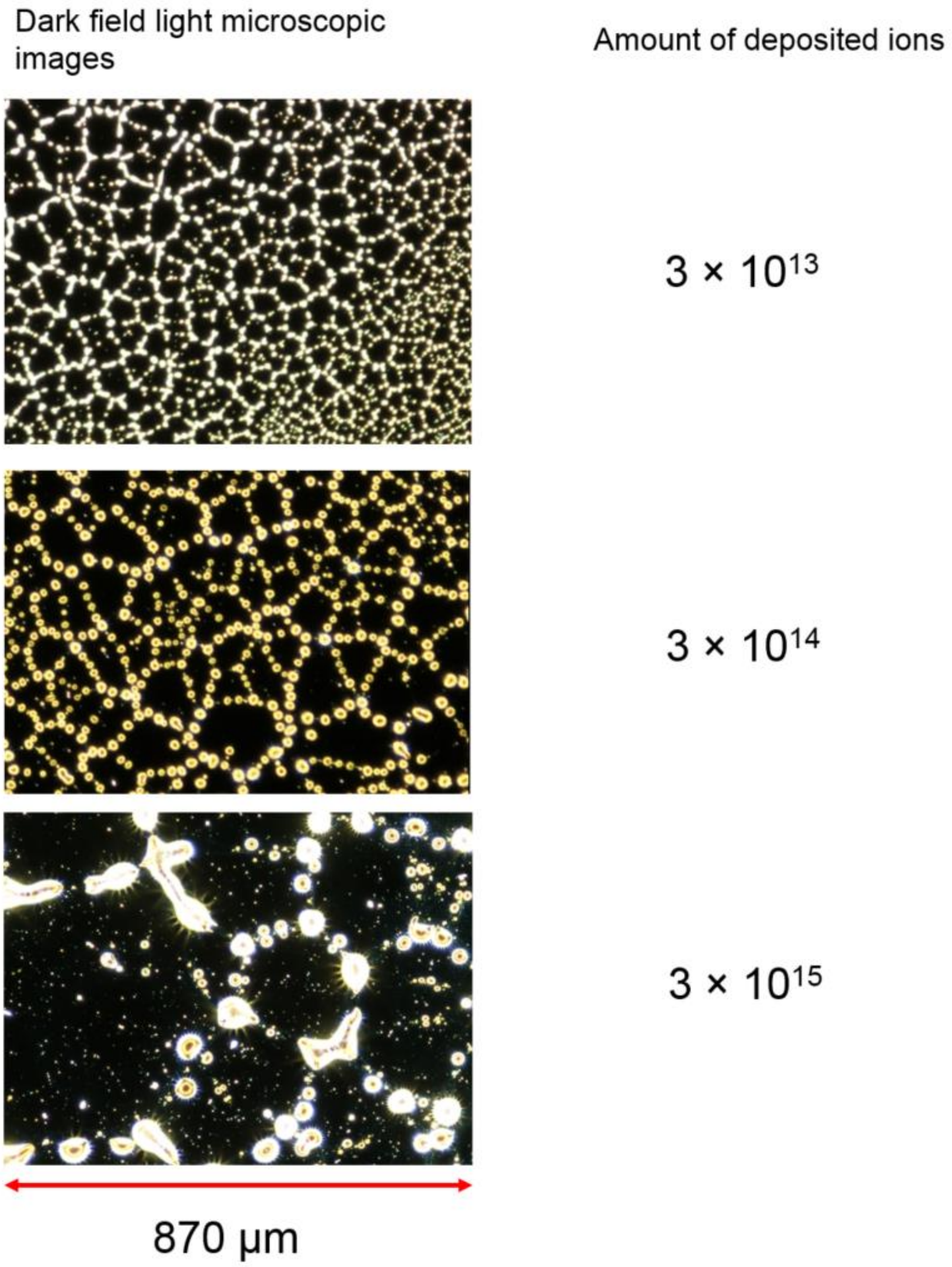

Supplementary Figure 6. Comparison of the droplet arrangement after dewetting of layers formed by soft landing of different amounts of $[B_{12}Cl_{12}]^{2-}$ showing substantially larger holes at higher coverages with droplets arranged at the edges.

Supplementary Note 5: IR investigations

Layers prepared by soft-landing of $[B_{12}X_{12}]^{2-}$ ions (X=F-I) onto SAMs were analyzed using *in-situ* infrared-reflection-absorption spectroscopy. The assignment of IR bands to adventitious hydrocarbons that bind to the surface is supported by comparison of the spectra for different X (Supplementary Figure 10). The growth of the hydrocarbon signals was found to be correlated to the growth of the ion signal: Slower deposition (lower current) did not result in a significant change of the signal ratios. An increase in the hydrocarbon bands in proportion to the ion signals was detected during deposition, see Supplementary Figure 7.

Measured IR signals are consistent with charge retention by the deposited anions, as shown in Supplementary Figure 8 for $[B_{12}F_{12}]^{2-}$. Note that $[B_{12}F_{12}]^{2-}$ has the lowest oxidation potential in the series of halogenated dodecaborates suggesting that this anion should lose an electron more efficiently than other $[B_{12}X_{12}]^{2-}$ species. Structures for $[B_{12}F_{12}]^{2-}$, $[B_{12}F_{12}]^{-}$ and $[B_{12}F_{12}]$ were optimized with DFT (PBE0/def2-tzvpp)[3] and frequency analyses were performed. The calculated spectra are compared with the experimentally obtained spectrum for deposited $[B_{12}F_{12}]^{2-}$ anions in Supplementary Figure 8. The same comparison for $[B_{12}Cl_{12}]^{2-}$ is shown in Supplementary Figure 9.

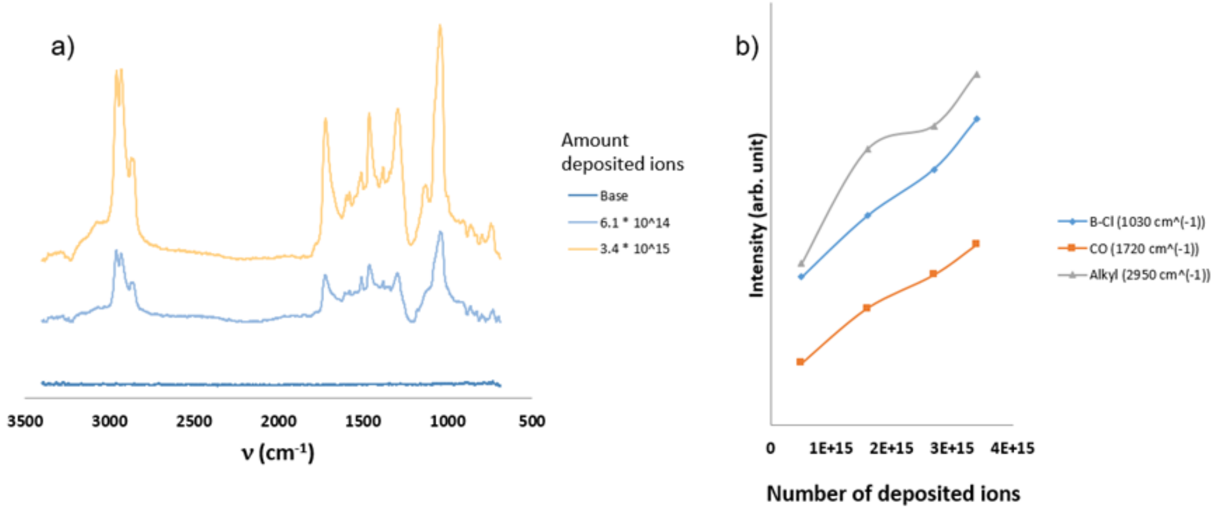

Supplementary Figure 7. a) IRRAS spectra measured during the deposition of $3.4*10^{15}$ ions of $[B_{12}Cl_{12}]^{2-}$, b) Plot of integrated peak intensities during deposition showing the growth of the hydrocarbon and anion bands during deposition. The clear onset to zero can be traced back to the detection limit and a change in the base line that usually occurs over time

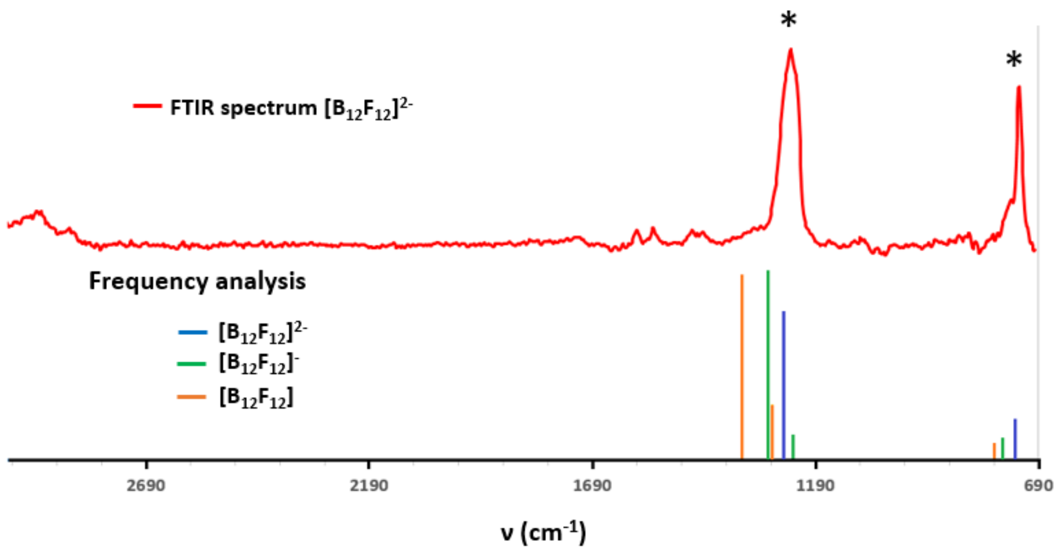

Supplementary Figure 8. Comparison of the experimentally measured $[B_{12}F_{12}]^{2-}$ IR signals after ion deposition and theoretically predicted frequencies for different charge states of the ion. $[B_{12}F_{12}]^{2-}$ signals are marked with a star. All other signals are assigned to adventitious hydrocarbons.

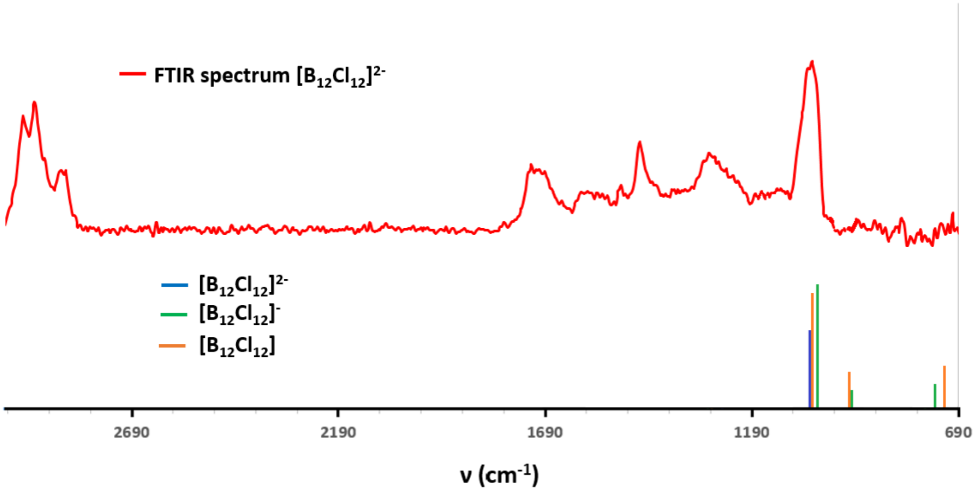

Supplementary Figure 9. Equivalent comparison as described in Supplementary Figure 8 for $[B_{12}Cl_{12}]^{2-}$.

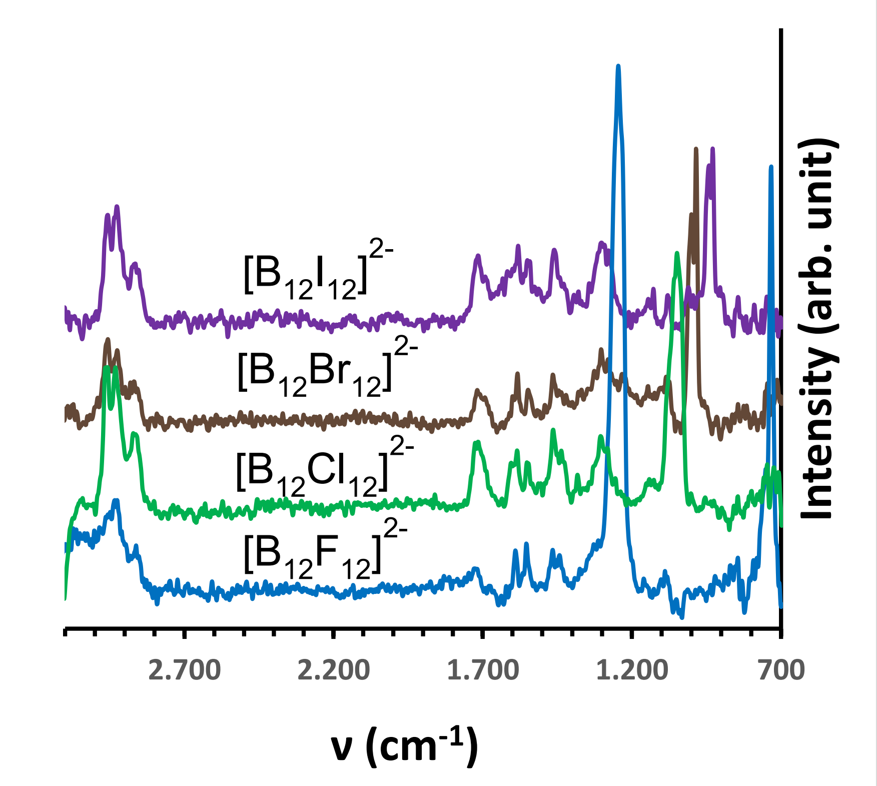

Supplementary Figure 10. IRRAS spectra detected during deposition of halogenated dodecaborates.

Supplementary Note 6: Mass spectrometry analysis of layer

Molecular formula assignments for the detected hydrocarbons are provided in Supplementary Table 1. Most of these molecular formulas are typical for phthalates. Phthalates are used as plasticizers. MS/MS experiments and comparison with MS/MS of a phthalate standard clearly confirmed this assignment, see example in Supplementary Figure 11 and 12. In fragmentation experiments, the $[M+Na]^+$ loses sequentially the ester backbone resulting in formation of a free diacid. Nano-DESI analysis of the $[B_{12}I_{12}]^{2-}$ layer resulted in the detection of the same hydrocarbons in positive ion mode that were detected for layers generated by depositing $[B_{12}Cl_{12}]^{2-}$shown in Figure 2b. In negative ion mode, a strong $[B_{12}I_{12}]^{2-}$ signal was detected.

Supplementary Table 1. Molecular formula assignments of the co-adsorbed hydrocarbons detected using nano-DESI high-resolution mass spectrometry in positive ion mode.

m/z	formula
273.167	$C_{12}H_{26}O_5Na$
331.209	$C_{15}H_{32}O_6Na$
441.298	$C_{26}H_{42}O_4Na$
455.313	$C_{27}H_{44}O_4Na$
469.323	$C_{28}H_{46}O_4Na$
585.376	$C_{33}H_{54}O_7Na$
599.392	$C_{34}H_{56}O_7Na$
613.407	$C_{35}H_{58}O_7Na$
627.423	$C_{36}H_{60}O_7Na$
641.439	$C_{37}H_{62}O_7Na$
873.621	$C_{53}H_{86}O_8Na$
887.637	$C_{54}H_{88}O_8Na$

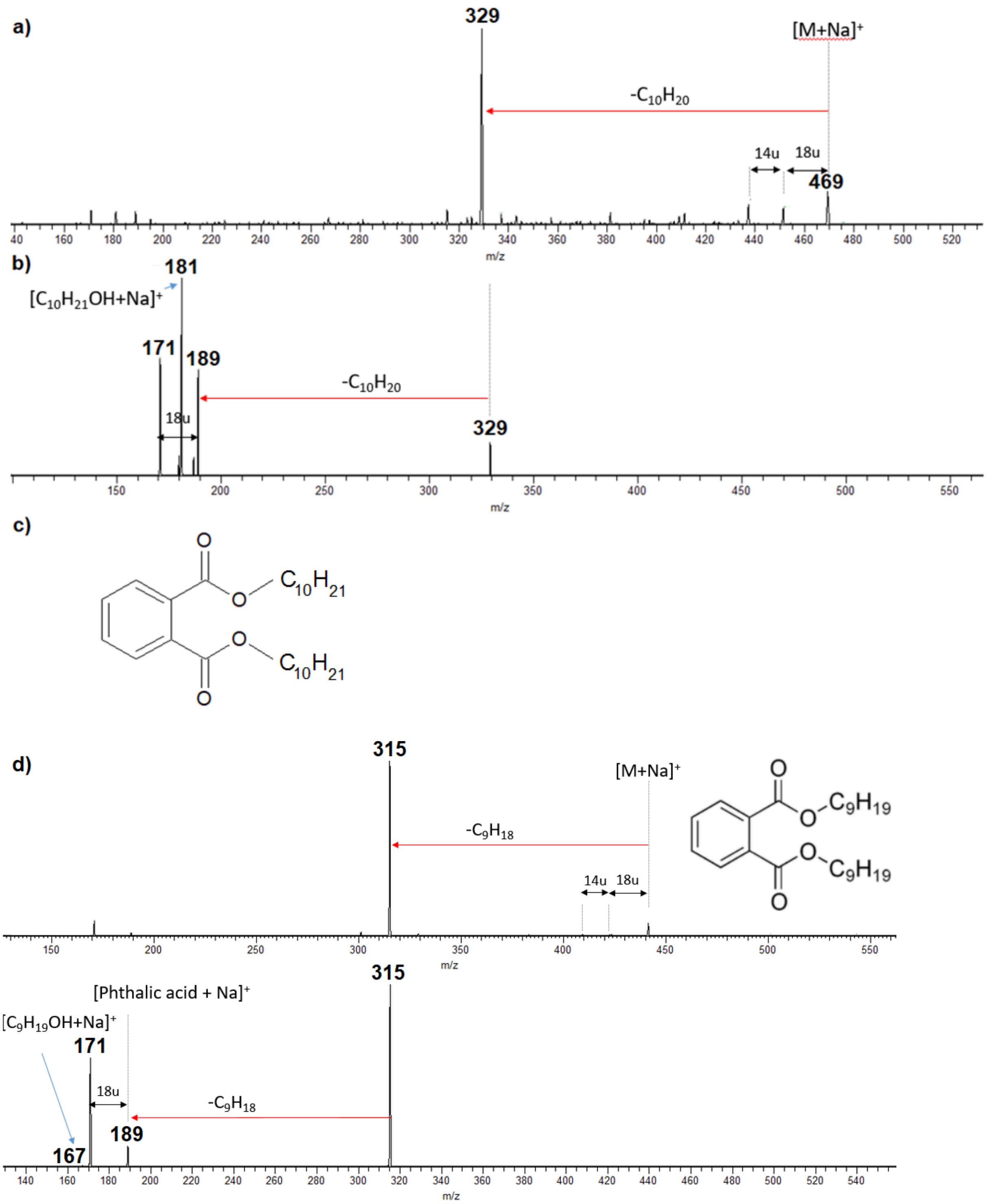

Supplementary Figure 11. Representative positive mode MS^2 a) and MS^3 b) spectra showing the fragmentation behavior of the hydrocarbons adsorbed into the layer and confirming their identification as phthalates. This fragmentation behavior was reproduced for a phthalate sample, c) The assigned structure, d) fragmentation behavior of the phthalate standard diisononyl phthalate.

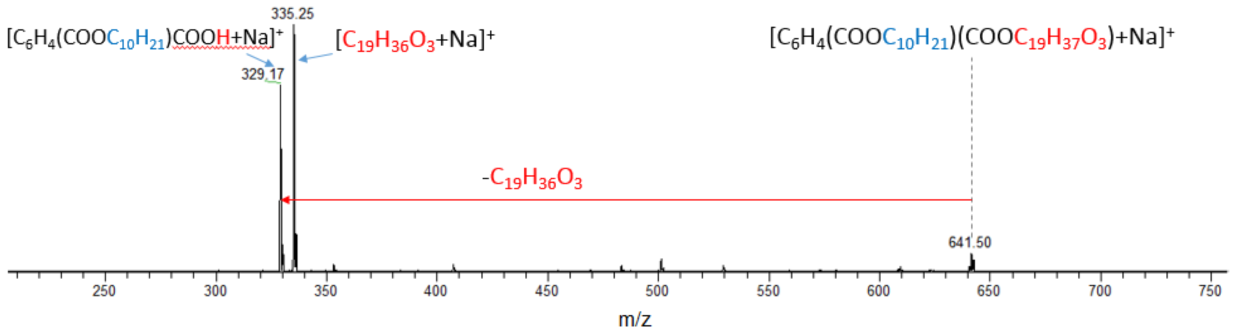

Supplementary Figure 12: Fragmentation behavior of one of the larger adventitious hydrocarbons. The secondary fragmentation pattern of the m/z 329 ion is identical to the fragmentation of the m/z 329 ion in the Supplementary Figure 11b.

Supplementary Note 7: XPS analysis

XPS chemical shifts indicate that carbonyl groups are present in the hydrocarbons, see Supplementary Figures 13,14. To clarify the nature of oxygen detected in the sample, peak fitting of the carbon XPS peak was performed to evaluate quantitatively the oxygen bound carbon, see Supplementary Figure 14. This analysis revealed that almost all detected oxygen is carbon bound. However, in dewetted layers, the oxygen content increased significantly (see boron to oxygen ratio in Supplementary Table 2), which was attributed to the intake of water. Note that the carbon signal cannot be considered here, since the layer is dewetted and signals from the underlying SAM contribute to these results as can be clearly seen from the large fluorine content.

Analysis of layers was performed on $[B_{12}I_{12}]^{2-}$ for comparison. In the case of the $[B_{12}I_{12}]^{2-}$ deposition, XPS elemental analysis indicated a C/B/O ratio of 55/12/10, which is very similar to the results listed in Table 1 for the $[B_{12}Cl_{12}]^{2-}$ layer.

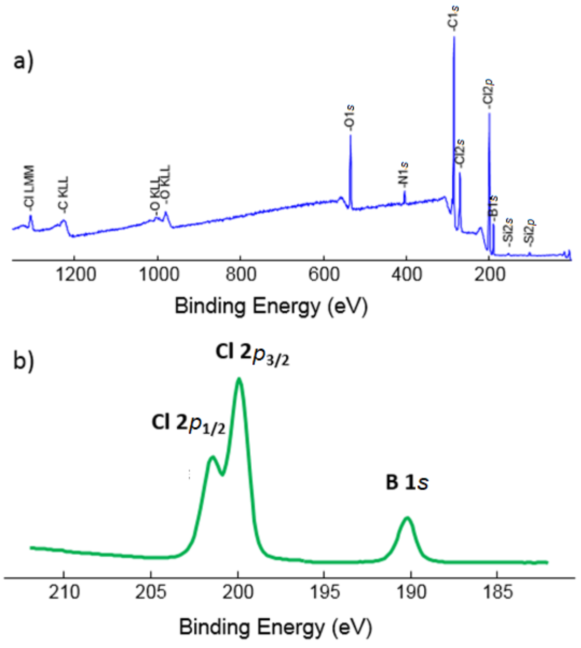

Supplementary Figure 13. a) Wide energy range XPS spectrum, b) High energy resolution XPS spectrum showing the boron signal c) XPS oxygen and d) carbon signals detected from the layer formed by $[B_{12}Cl_{12}]^{2-}$ deposition.

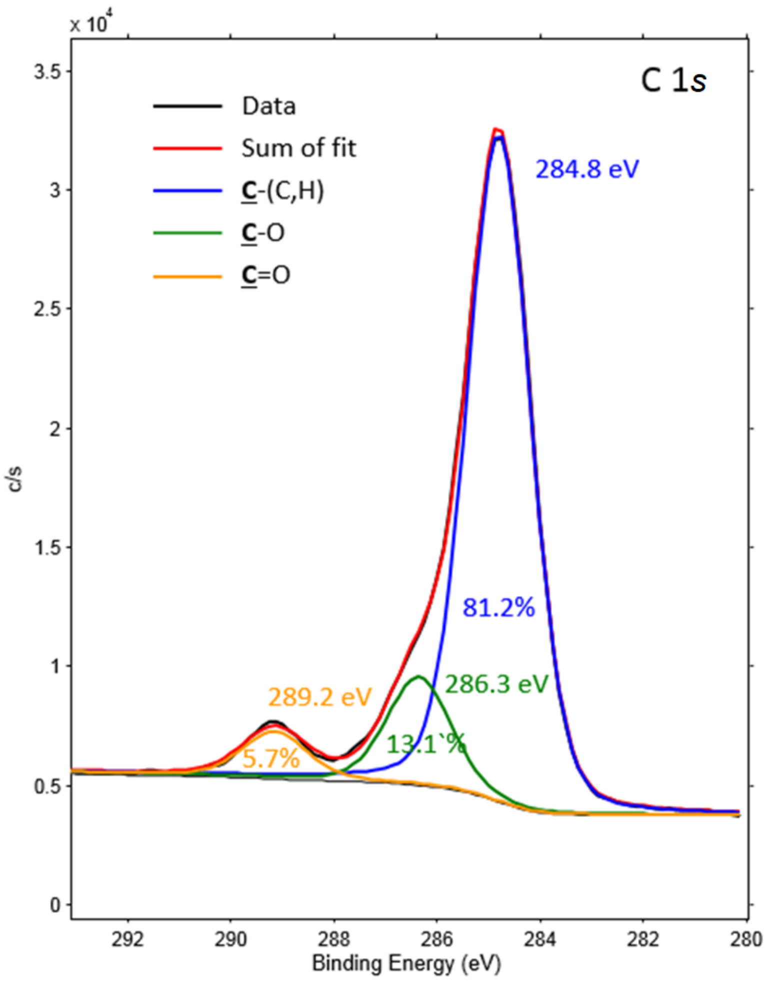

Supplementary Figure 14. Peak fitting of the carbon XPS spectrum in Supplementary Figure 13d to distinguish between different contributions of oxygen bound carbon and CH_x groups.

Supplementary Table 2: XPS elemental analysis (atomic concentrations in %) of a dewetted layer. Note that the SAM contributes to the measured signals. Both measurements were done at different positions of the same layer.

Measurement	B(1*s*)	C(1*s*)	O(1*s*)	F(1*s*)	Cl(2*p*)	Au(4*f*)
1	2.9	29.6	7.3	26.8	3.0	30.4
2	2.6	30.3	8.7	22.1	3.2	33.1

Supplementary Note 8: NMR analysis

A $[B_{12}I_{12}]^{2-}$ layer with coverage of roughly $6{\cdot}10^{15}$ ions was dissolved in deuterated methanol. The ^{11}B NMR showed the corresponding signal clearly shifted towards the ^{11}B signal detected from the dissolved $[B_{12}Cl_{12}]^{2-}$ layer, see Supplementary Figure 15, in agreement with the literature.[4]

We note that an additional lower intensity ^{11}B NMR signal (+18 ppm) was detected during our investigation of the dissolved $[B_{12}Cl_{12}]^{2-}$ layer. This chemical shift corresponds well to the ^{11}B chemical shift of the boric acid standard used as a reference, but species such as $(RO)_2BX$ (X=halogen) have also been observed at similar ranges of the chemical shift. The additional resonance may have formed as a decomposition product over time in these samples. The dissolved layers were stored prior to NMR investigations for 9 months. The $[B_{12}I_{12}]^{2-}$ layer was stored prior to NMR investigations only for 3 weeks and showed the same signal with more than an order of magnitude weaker relative intensity. All other analytical methods (like XPS) which have been performed in the time frame of minutes to days after deposition, did not indicate the presence of any other boron-containing molecules.

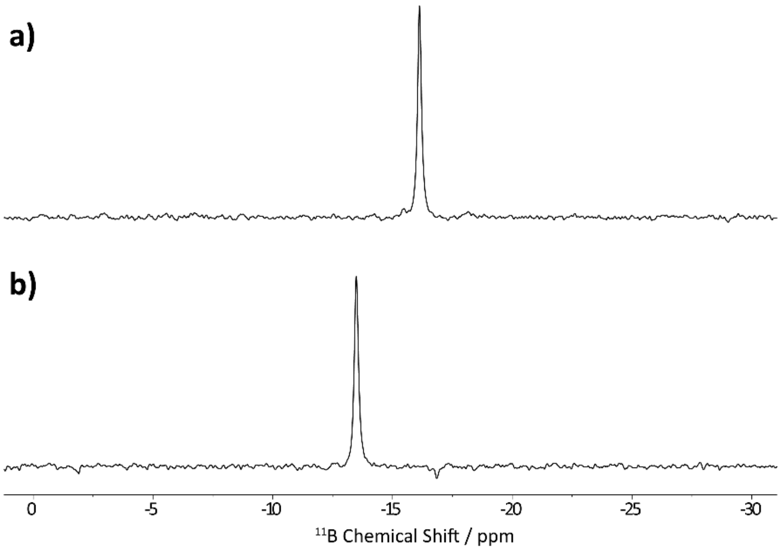

Supplementary Figure 15. ^{11}B NMR signals of deposited anions detected in dissolved soft landed layers of $[B_{12}I_{12}]^{2-}$ (top) and $[B_{12}Cl_{12}]^{2-}$ (bottom). Spectra were referenced to an external sample of 189 mM boric acid in D_2O, pD 7.8 (pD = pH + 0.4), assigned as +18.00 ppm. A left-shift of the FID by 60 points (300 µs) was performed to improve the baseline and remove the high background signal emanating from both the borosilicate probe insert and NMR tube. Other post-acquisition processing included backward linear prediction using 32 coefficients and 256 basis points, exponential multiplication of the FID (22 Hz line broadening), and baseline correction.

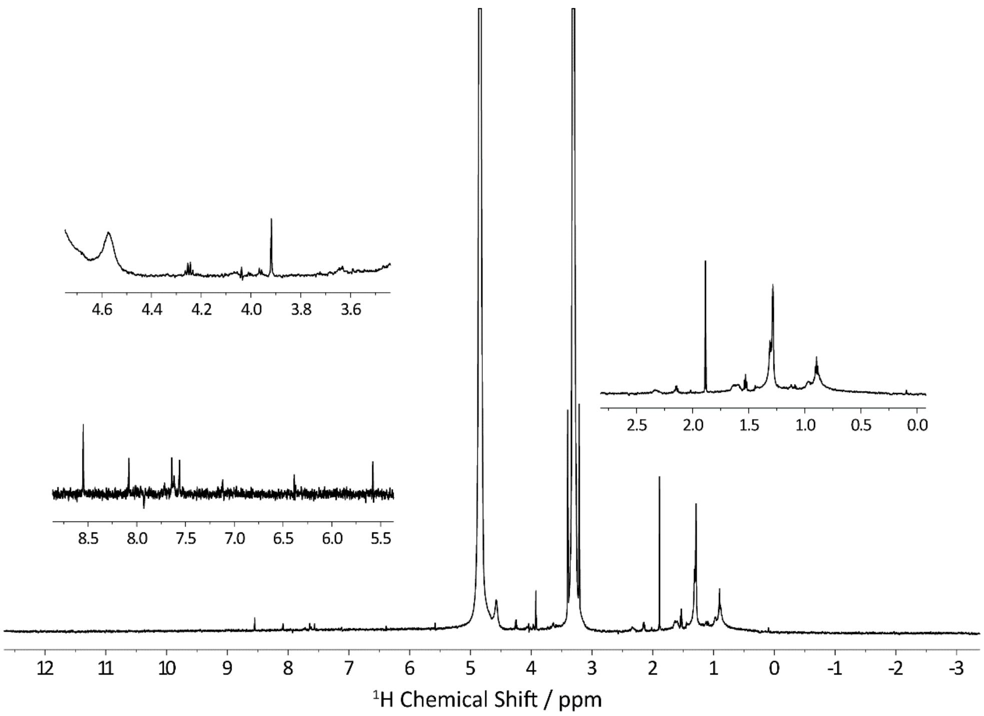

Supplementary Figure 16. ^{1}H NMR spectrum of the dissolved $[B_{12}Cl_{12}]^{2-}$ layer in deuterated methanol (CD_3OD). Regions of interest have been expanded for greater detail. Note the dominant signals at 3.3 and 4.9 ppm are originating from the solvent.

Supplementary Note 9: XRD-analysis

In an effort to obtain information on long range order, micro-XRD investigations on droplets were performed. No polycrystalline order was detected in the droplets by XRD. We did not detect any peaks that may correspond to the material in the 5-100 degrees 2-Theta range (using Cr $K_{\alpha,\lambda}$ radiation; 2.2897 Å). This observation further supports the liquid nature of the material.

Supplementary Note 10: Morphology of the $[B_{12}F_{12}]^{2-}$ layer and droplets

The morphology of layers generated by soft-landing of $[B_{12}F_{12}]^{2-}$ and $[B_{12}Cl_{12}]^{2-}$ onto FSAM in the initial and final stage of dewetting are compared in Supplementary Figure 17. It is obvious that the layer itself is more grainy in the case of $[B_{12}F_{12}]^{2-}$. AFM results indicate a solid character of the droplets formed in the case of $[B_{12}F_{12}]^{2-}$, see Supplementary Figure 18. Line profiles in Supplementary Figure 19 are shown for droplets formed by dewetted $[B_{12}Br_{12}]^{2-}$ and $[B_{12}I_{12}]^{2-}$ layers.

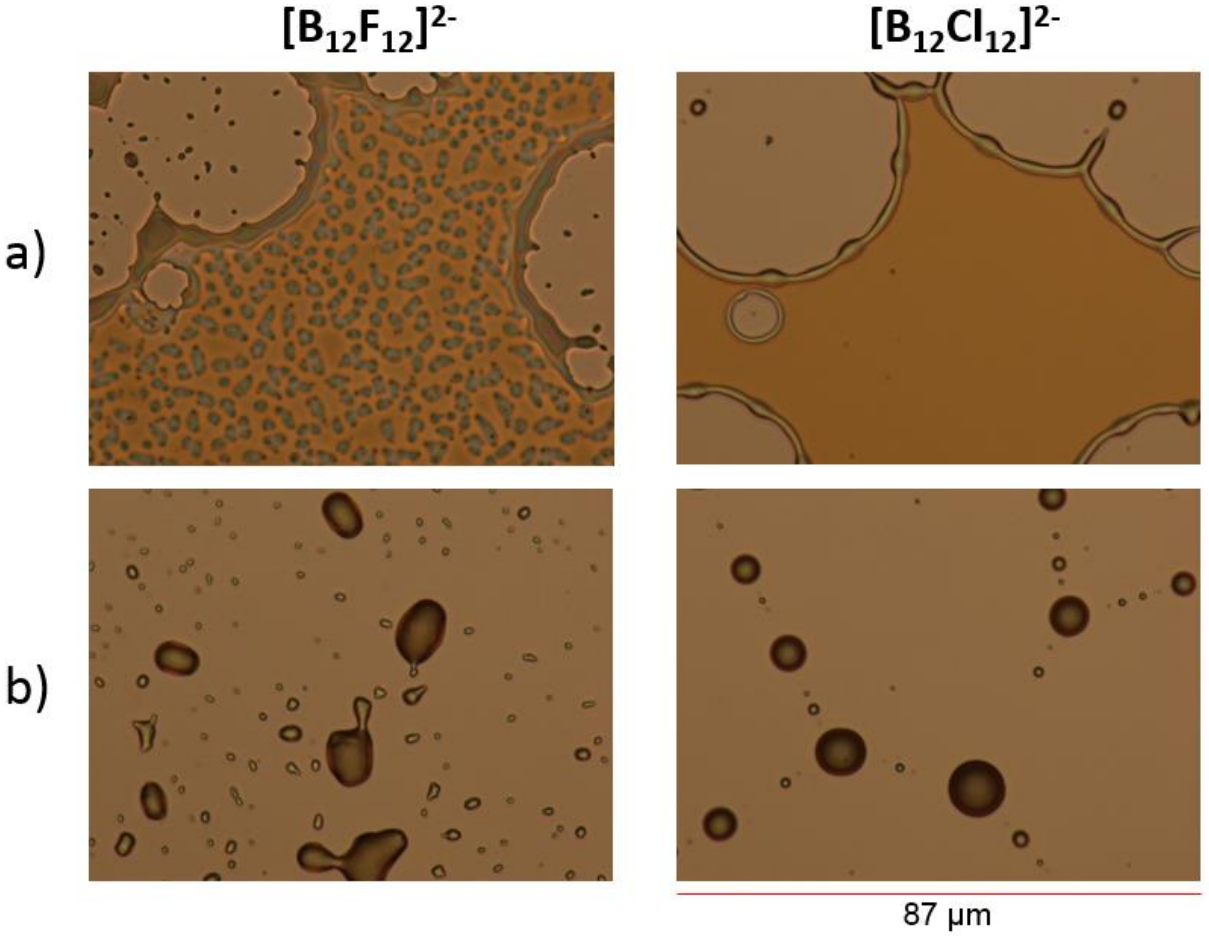

Supplementary Figure 17. Bright field optical microscope images of layers generated by deposition of $[B_{12}F_{12}]^{2-}$ and $[B_{12}Cl_{12}]^{2-}$ layers on FSAM a) during the initial stage of dewetting and b) after final droplet formation.

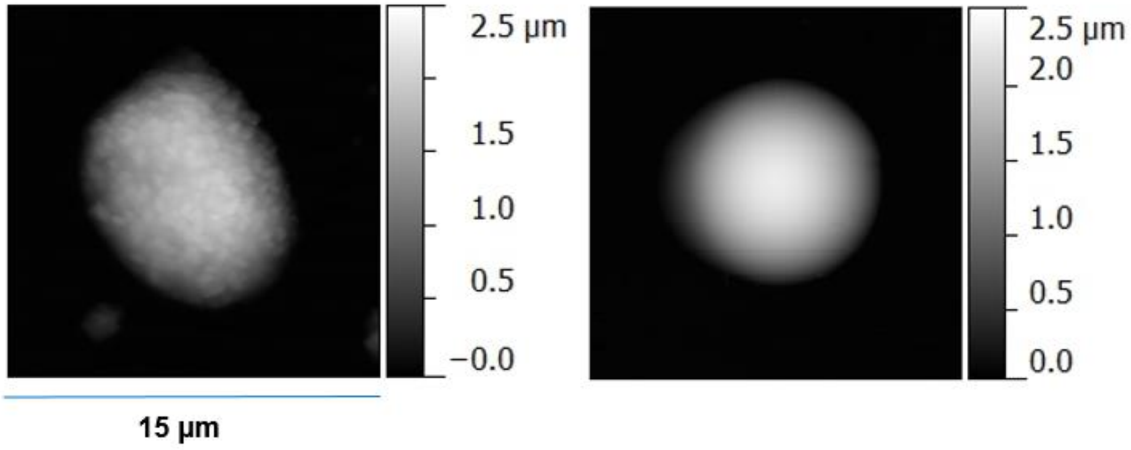

Supplementary Figure 18. AFM images shown in Figure 3 in grey scale.

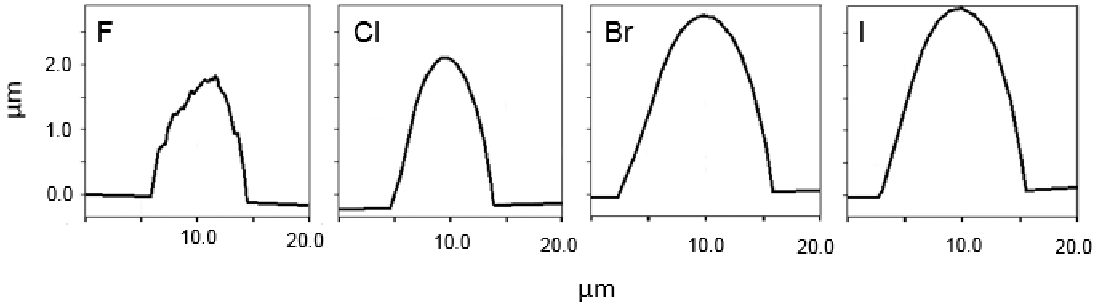

Supplementary Figure 19. Line profiles (AFM) of droplets after dewetting of $[B_{12}X_{12}]^{2-}$ based layers after dewetting.

Supplementary Note 11: Vacuum drying

We performed vacuum drying of a $[B_{12}Cl_{12}]^{2-}$ layer after the initial dewetting stage. Straight circular holes as shown in Figure 3 in the manuscript appeared in the layer prior to the process. Subsequent storage for one week in the soft landing instrument under vacuum resulted in the deliquescence of the straight hole borders showing that the driving force for the dewetting process has been removed. The hole borders did not recover, but instead new holes were formed in the areas of intact layer after some time under ambient conditions.

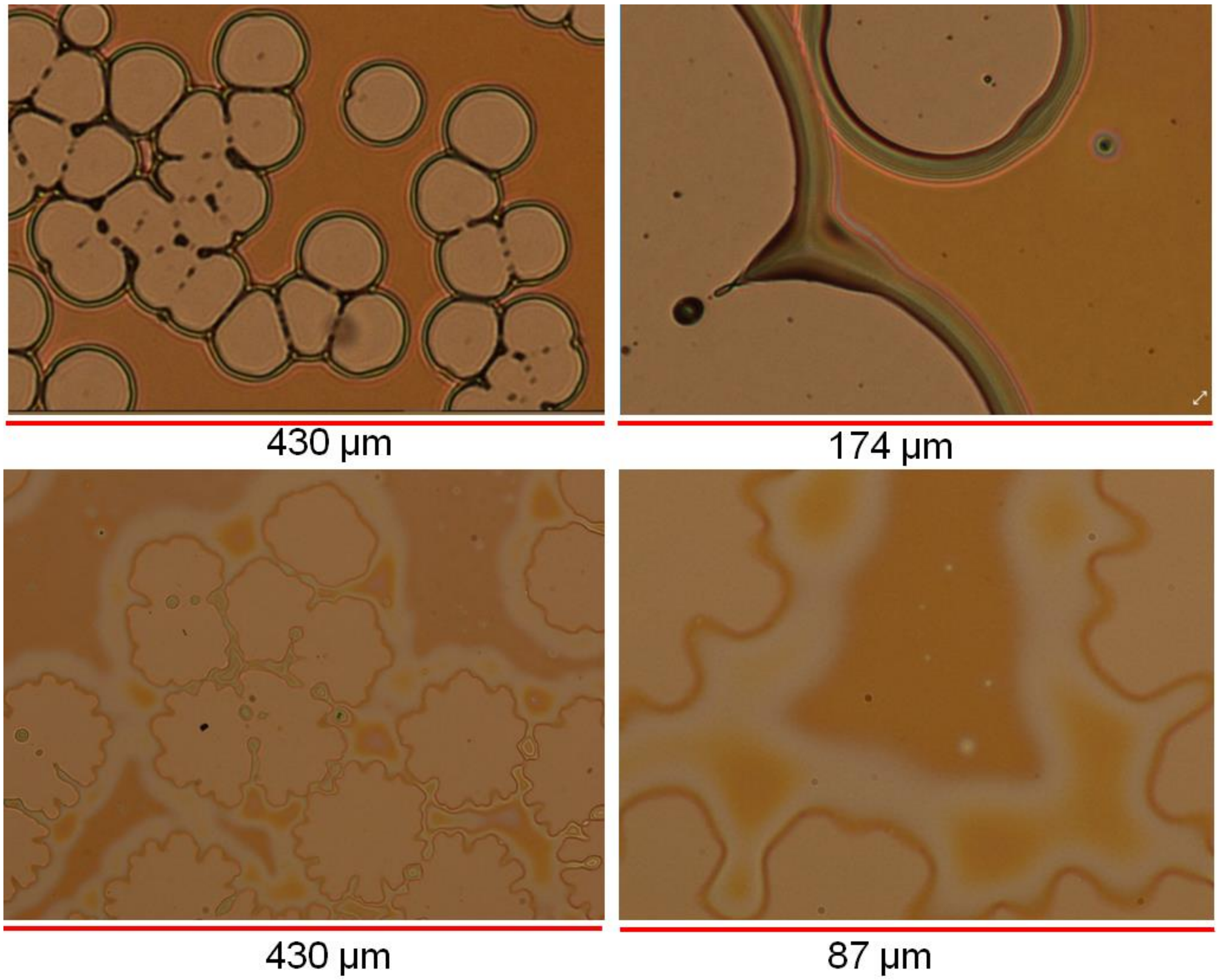

Supplementary Figure 20: Comparison: Top: Hole borders in the initial step of dewetting (left: low magnification, right high magnification), bottom: Hole borders after vacuum drying.

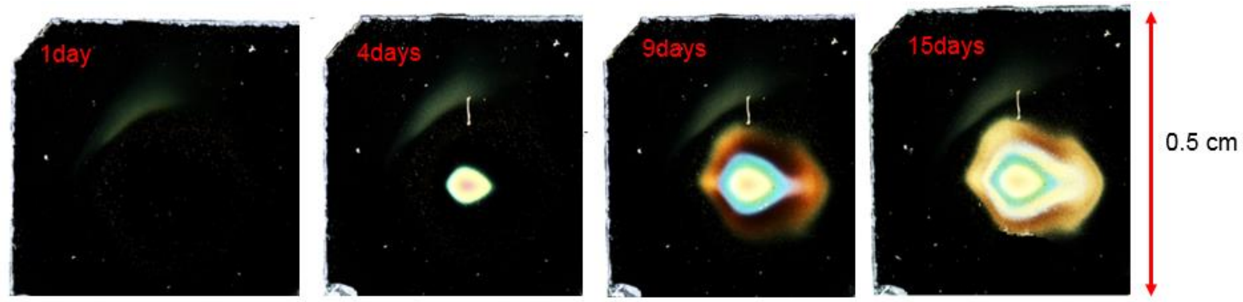

Supplementary Figure 21. Dark field optical microscope images of a layer generated by deposition of $[B_{12}I_{12}]^{2-}$. The surface was stored at ambient conditions.

Supplementary Note 12: Dewetting of $[B_{12}Br_{12}]^{2-}$ and $[B_{12}I_{12}]^{2-}$ based layers

During the induced dewetting of layers generated by deposition of $[B_{12}Br_{12}]^{2-}$ and $[B_{12}I_{12}]^{2-}$, it was observed that $[B_{12}I_{12}]^{2-}$ layers needed roughly ten times longer to reach the final stage of dewetting (formation of droplets) than $[B_{12}Br_{12}]^{2-}$. Dewetting was also induced by breathing or blowing wet nitrogen over the target. This resulted in the same visual effect. Eight times breathing was necessary to dewet a $[B_{12}Br_{12}]^{2-}$ layer ($5*10^{14}$ ions), while under same conditions 98 breathes were necessary to form droplets in the corresponding $[B_{12}I_{12}]^{2-}$ layer. Images obtained during dewetting of a $[B_{12}Br_{12}]^{2-}$ layer are shown in Supplementary Figure 22. An investigation of the long term development of a $[B_{12}I_{12}]^{2-}$ layer that was stored under ambient conditions is documented in Supplementary Figure 21. The very slow water-intake results in changes in the optical appearance over several weeks.

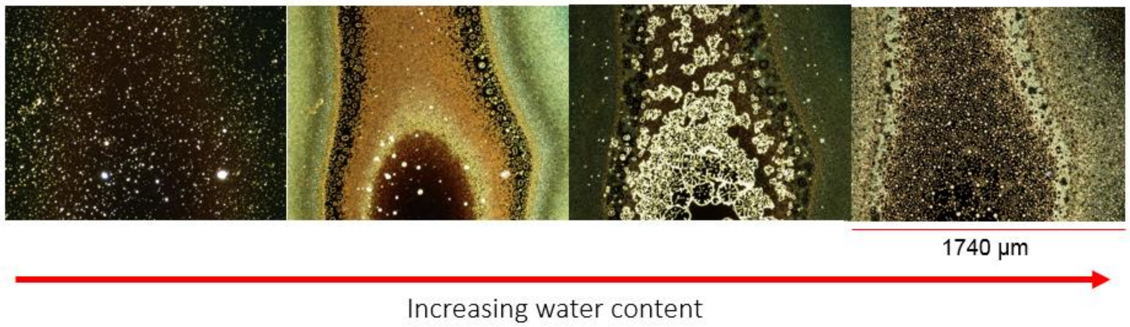

Supplementary Figure 22. Dark field optical microscope images obtained during water induced dewetting of a $[B_{12}Br_{12}]^{2-}$ surface.

Supplementary Note 13: Layers on HSAM

The morphologies of layers under ambient conditions (Supplementary Figure 23) and after induced dewetting for X=Br, I (Supplementary Figure 24) on FSAM and HSAM are compared. On the FSAM droplets are formed while on the HSAM the morphology is very different.

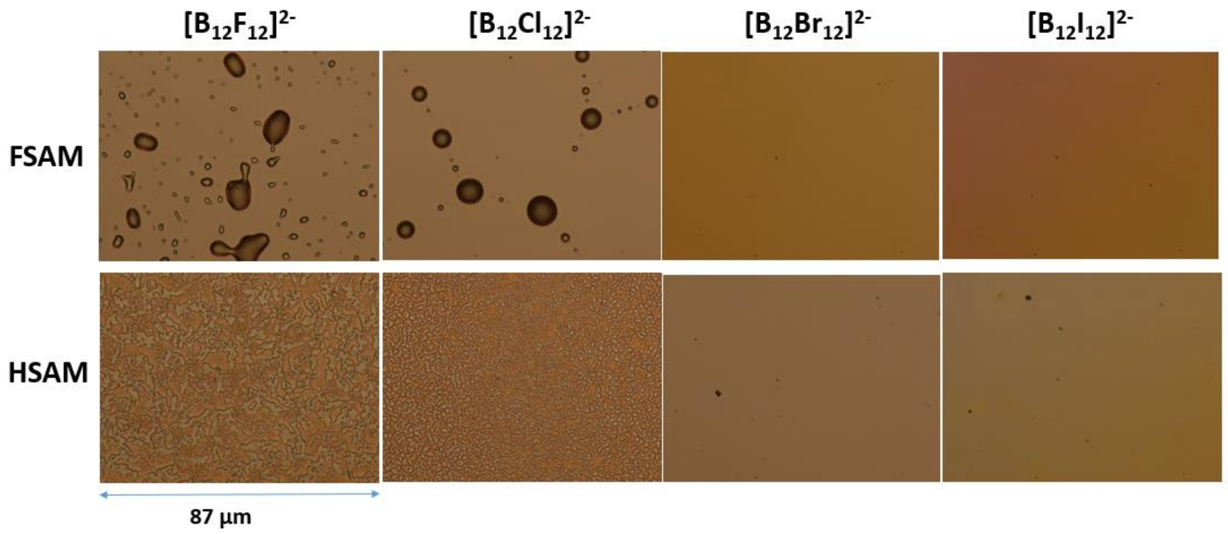

Supplementary Figure 23. Morphologies of layers generated by deposition of $[B_{12}X_{12}]^{2-}$ ions under ambient conditions on FSAM and HSAM.

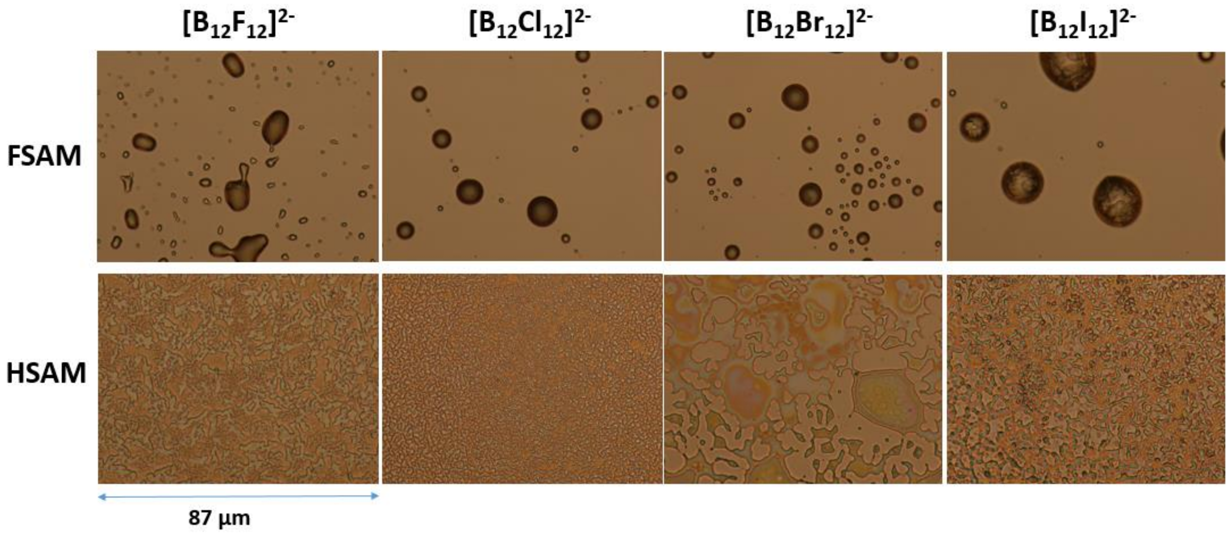

Supplementary Figure 24. Same comparison like in Supplementary Figure 23 after induced dewetting of the $[B_{12}Br_{12}]^{2-}$ and $[B_{12}I_{12}]^{2-}$ layer.

Supplementary Note 14: Phthalate composition

To investigate the reproducibility of the obtained results, layers were generated after the soft landing instrument was taken apart and cleaned, seals replaced, a pump repaired and the pump oil changed. Although the composition of the phthalates changed in terms of relative ratios (see Supplementary Figure 25a), the macroscopic behavior was well reproduced (Supplementary Figure 25b). The $[B_{12}Cl_{12}]^{2-}$ layers showed the previously described dewetting while $[B_{12}I_{12}]^{2-}$ layers were stable during this time frame. It was also possible to substitute the distribution of phthalates by one defined phthalate, see supplementary note 15.

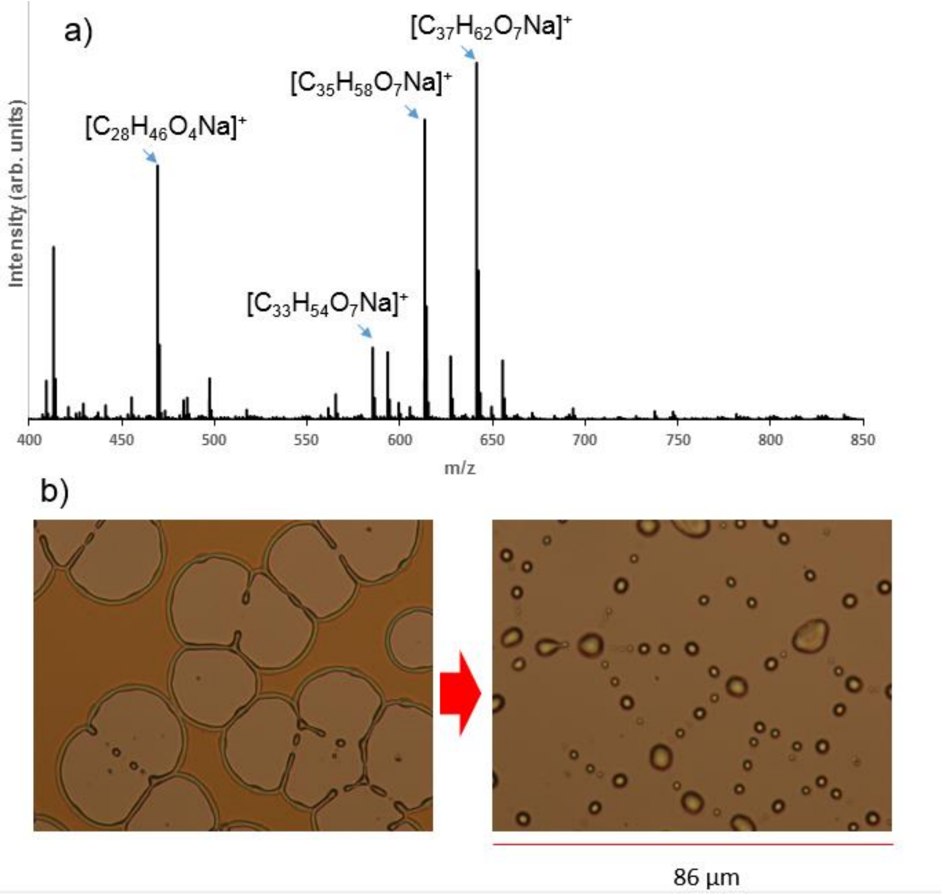

Supplementary Figure 25. a) ESI, positive mode, mass spectra of a $[B_{12}Cl_{12}]^{2-}$ based layer generated after significant changes to the instrument and pumps were performed. The layer behavior is well reproduced as shown in b.

Supplementary note 15: Codeposition of a defined phthalate

By introduction of a liquid phthalate into the soft-landing instrument with base pressure 10^{-8} mbar and heating of the reservoir to 80-120°C, we could substitute the distribution of phthalates almost completely with pure diisodecyl phthalate. The base pressure increased during the experiment by one to two orders of magnitude. A residual gas analyzer mass spectrum is shown in Supplementary Figure 26, which shows typical signals of diisodecylphthalates in the measurable mass range (see inserted reference spectrum from NIST Chemistry webbooks for comparison). Supplementary Figure 27 shows mass spectrometric analysis of the layer and optical microscopy of the dewetted layer.

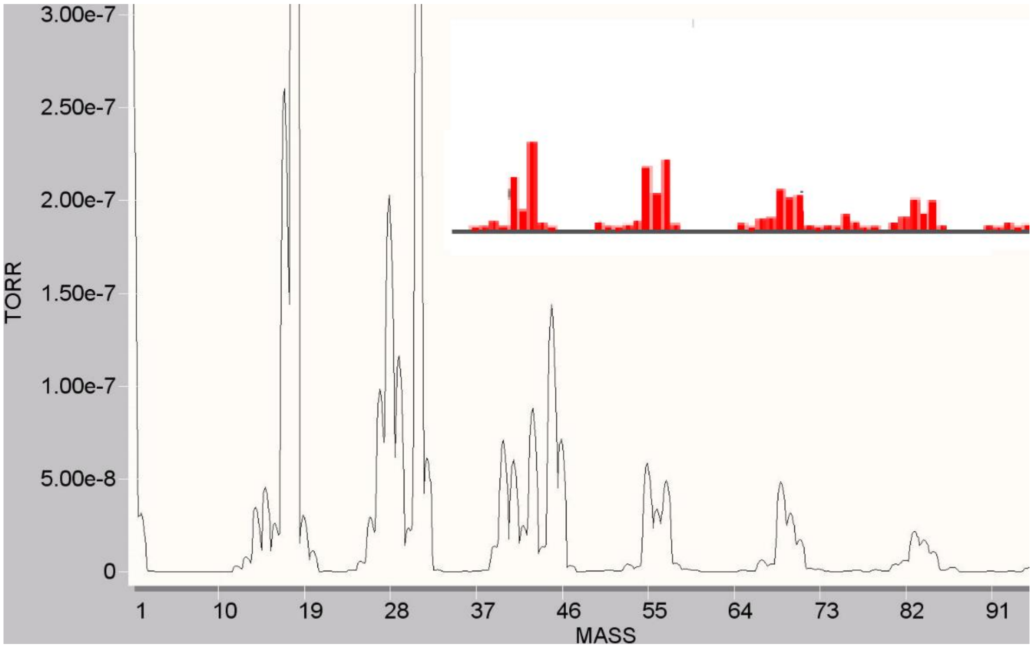

Supplementary Figure 26: Screenshot of a residual gas analyzer mass spectrum during the deposition of $[B_{12}Cl_{12}]^{2-}$ and heating of condensed phthalate inside the vacuum chamber.

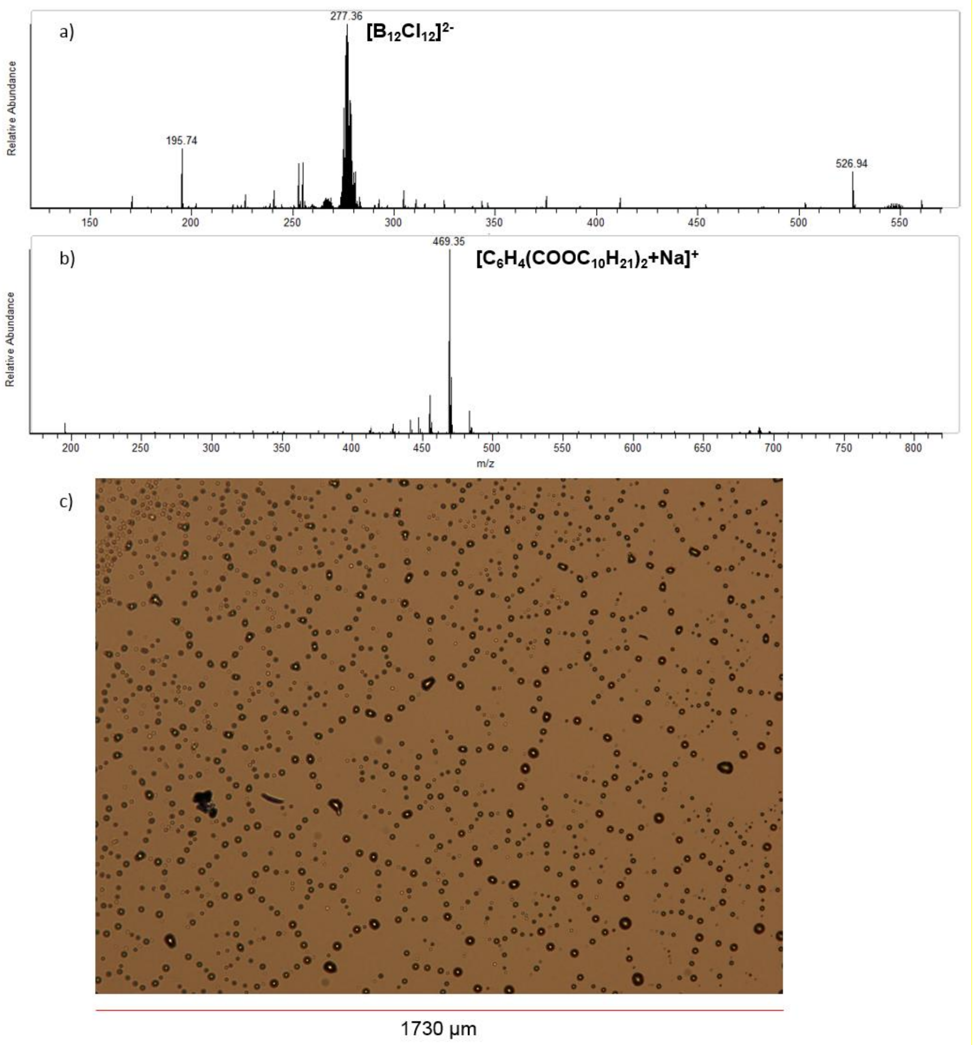

Supplementary Figure 27. a) negative and b) positive ESI-MS spectra of the dissolved layer after condensed diisodecyl-phthalate was introduced into the ion soft-landing instrument and heated to around 150˚C. The layer behavior was well reproduced. Figure c shows an image after dewetting.

Supplementary Note 16: Glycine codeposition experiment

Experiment 1: Partial substitution

For partial substitution of the adventitious hydrocarbons with glycine, we put solid glycine powder on a metal flange next to the gold surface to be used for deposition and heated it up in the vacuum chamber to 70˚C. The base pressure of $8*10^{-5}$ Torr determined by an ion gauge did not increase measurably. IR spectra measured before ion deposition showed that glycine is not deposited in any detectable amounts via chemical vapor deposition on the surface under these conditions. However, during ion deposition, the IR spectrum changed considerably compared to experiments without glycine. Still, the IR bands of the adventitious hydrocarbons were observed, see Supplementary Figure 28c. However, new IR bands attributed to glycine were clearly present in the spectrum.

Experiment 2: Full Glycine substitution

For full substitution of glycine we performed ion soft-landing in another apparatus with a lower base pressure (1×10^{-8} mbar). A glycine reservoir was heated to roughly 200˚C and the vapor was introduced into the deposition chamber via a heated leak valve. The reservoir was heated slowly in the timeframe of hours up to this temperature because only at 200˚C glycine could be detected by a residual gas analyzer attached to the main chamber (increased *m/z* 30 signal, the predominant species in the electron impact spectrum of glycine). This was accompanied by a pressure increase of two orders of magnitude in the deposition chamber. The experimental setup is similar to previously described instruments.[5] The $[B_{12}Cl_{12}]^{2-}$ ions were deposited under these conditions. Nano-DESI analysis was performed in the middle of the deposited spot. Mass spectra in the negative mode showed exclusively $[B_{12}Cl_{12}]^{2-}$, while in the positive mode only *m/z* 76 and 98 were detected. These signals are assigned to protonated glycine and glycine as a sodium adduct.

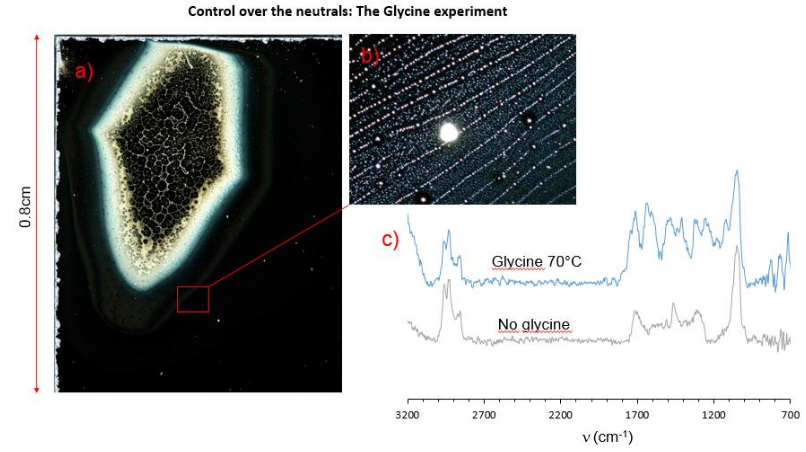

Supplementary Figure 28. a) dark field image of the deposited layer obtained from deposition of $[B_{12}Cl_{12}]^{2-}$ in the presence of glycine heated up to 70˚C, b) optical appearance of the borders in dark field images, c) comparison of IR spectra of deposited layers with and without glycine.

Supplementary Note 17: Electron beam induced tips analysis before and after methanol washing.

Electron beam induced tips were imaged using atomic force microscopy (AFM) in contact mode for the initial state and tapping mode for the washed state. A single tip was chosen for high-resolution analysis, shown in Supplementary Figure 29. Because the tip is asymmetric, line profiles across the tip were performed in two directions, along the widest and narrowest dimensions of the post-washed tip. The position of the profile was chosen to maximize the tip height. The asymmetry of the features is not likely an AFM imaging artifact due to the relatively small aspect ratio, especially for the initial state where the asymmetry is more prominent.

Full widths at half maxima drop after washing from 1.1 µm to 0.3 µm in Supplementary Figure 29c and 0.9 µm to 0.2 µm in Supplementary Figure 29d.

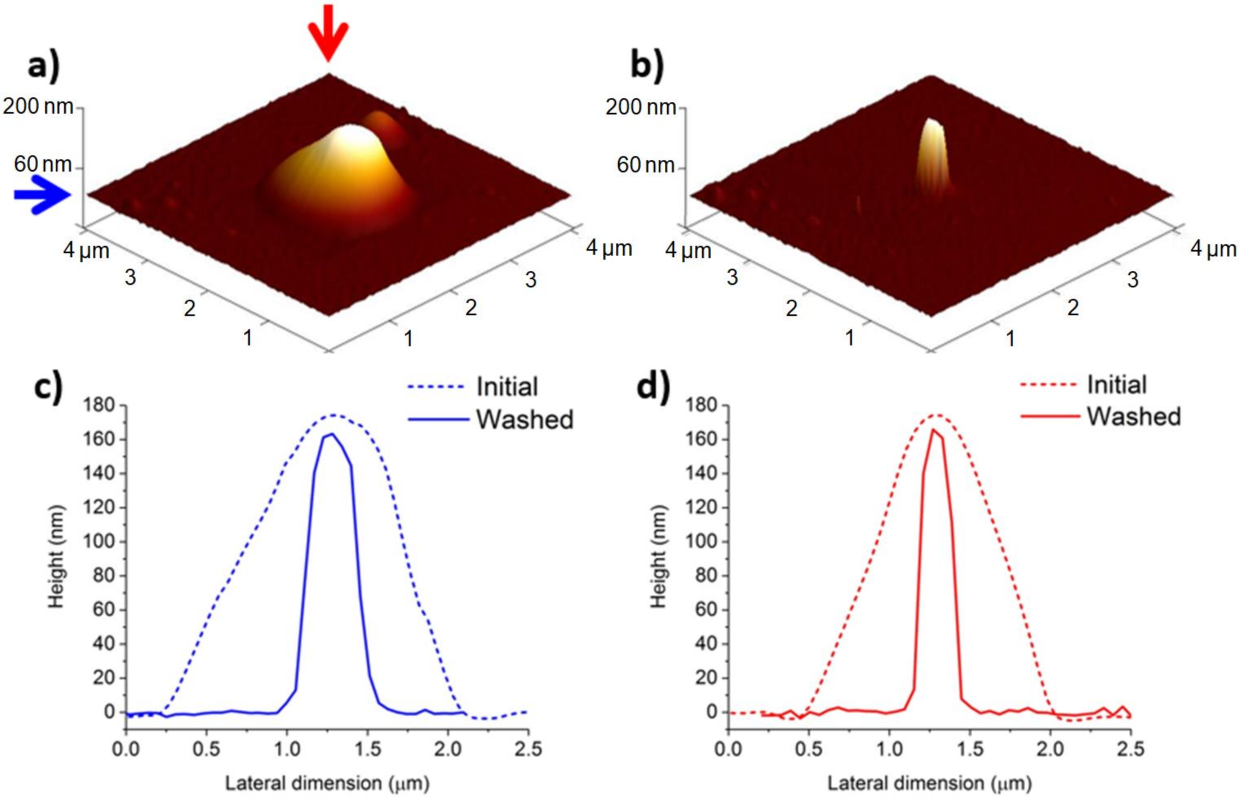

Supplementary Figure 29. 3-D representations of AFM topographic images of an electron beam-induced tip coated from a material front during dewetting a) before and b) after washing in methanol. c,d) Line profiles parallel to the colored arrows in a).

Supplementary Note 18: Binding of water and diisodecylphthalate to $[B_{12}Cl_{12}]^{2-}$

The binding energy of a diisodecyl-phthalate molecule and a water molecule was estimated using the DFT method B3LYP/def2-tzvppd[6] including dispersion forces[7]. We note that our investigation does not include all possible conformations of the organic alkyl chains of the phthalates. The binding energies should be considered as rough estimations. The optimized geometries are shown in Supplementary Figures 30-32 and the evaluated binding energies are 10.3 kcal/mol for $[B_{12}Cl_{12}+H_2O]^{2-}$, 29.1 kcal per mol for $[B_{12}Cl_{12}+C_{28}H_{46}O_4]^{2-}$ and 51.4 kcal per mol for $[B_{12}Cl_{12}+(C_{28}H_{46}O_4)_2]^{2-}$ (corresponding to the attachment of both phthalate molecules).

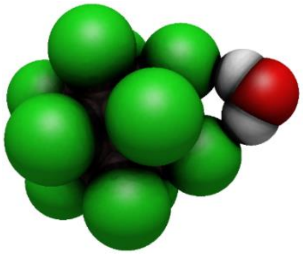

Supplementary Figure 30. Optimized geometry of $[B_{12}Cl_{12}+H_2O]^{2-}$.

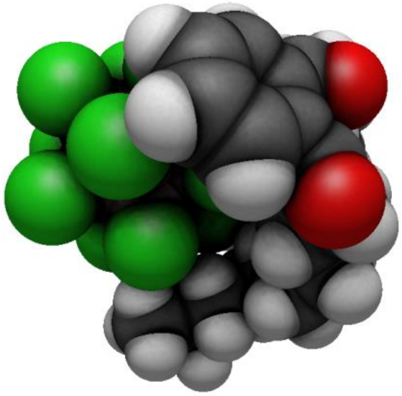

Supplementary Figure 31. Optimized geometry of $[B_{12}Cl_{12}+C_{28}H_{46}O_4]^{2-}$.

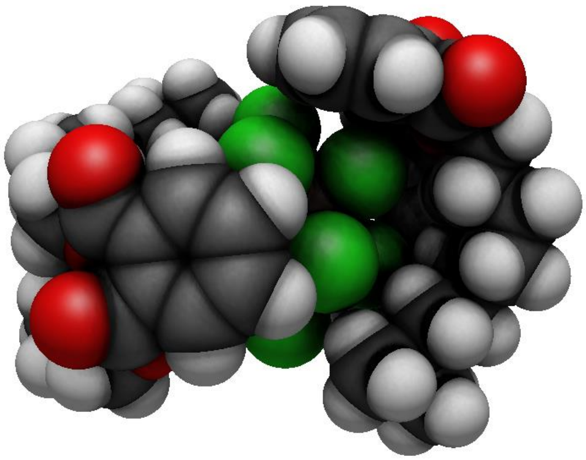

Supplementary Figure 32. Optimized geometry of $[B_{12}Cl_{12}+(C_{28}H_{46}O_4)_2]^{2-}$.

Supplementary Note 19: Correlation between phthalate contact angle and final morphology on surfaces

Droplets of diisodecyl-phthalate (0.5 µL) were placed on 3 different surfaces. As can be clearly seen from Supplementary Figure 33a, phthalates have a high contact angle on FSAM, a smaller contact angle on HSAM and wet the surface to a considerable extent on the unmodified gold surface. This behavior can be clearly correlated to the final stage of the layer after long exposure to environmental conditions (see Supplementary Figure 33c). On FSAM droplets are formed, on HSAM dewetting occurs, but no comparable free surface areas are formed and on an unmodified gold surface, the layer showed no visible morphological change.

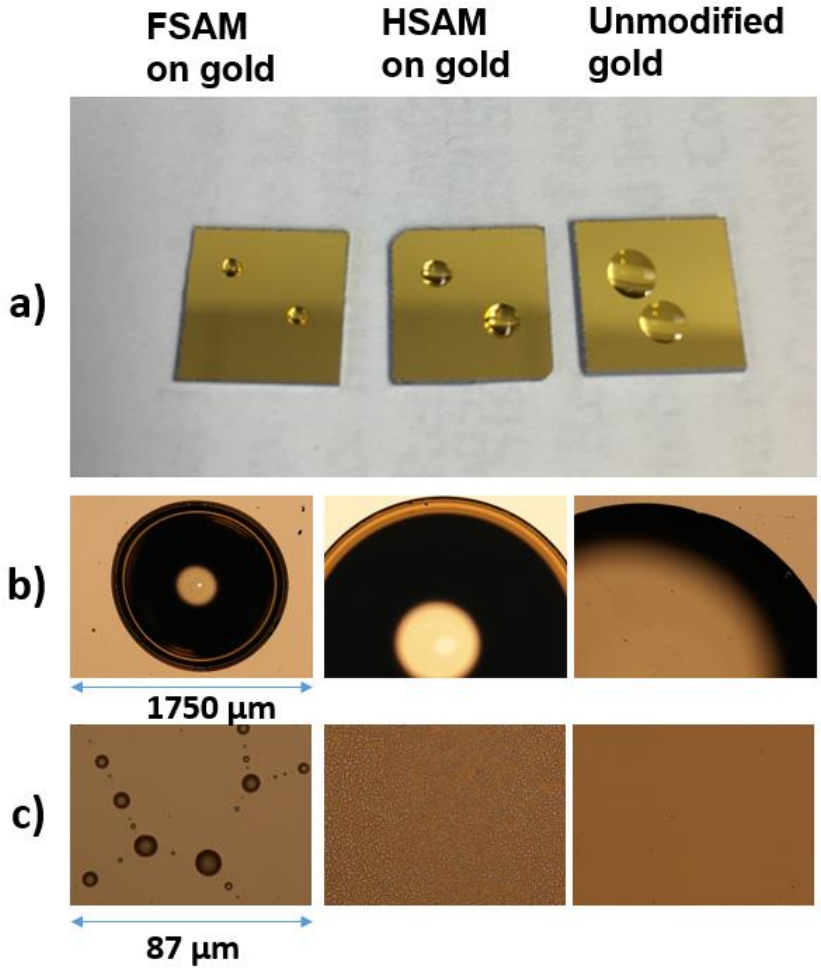

Supplementary Figure 33. a) Photo of 3 different surfaces (FSAM, HSAM, unmodified gold surface) after droplets of 0.5 µL diisodecylphthalate were placed on the surface, b) light microscopy images of the droplets, c) light microscopic images of the final stage of a $[B_{12}Cl_{12}]^{2-}$ based layer on the different surfaces.

Supplementary Note 20: Binding of water to $[B_{12}F_{12}]^{2-}$ and $[B_{12}I_{12}]^{2-}$ in comparison.

Supplementary Figure 34 schematically shows the disposition of a water molecule in close contact with the halogen shell of $[B_{12}F_{12}]^{2-}$ and $[B_{12}I_{12}]^{2-}$. The overall orientation of water molecules is governed by dipole-ion interactions between the water dipole and the negative charge of the boron clusters. This leads to the favorable attractive interaction of the water hydrogens with the negative fluorine shell in of $[B_{12}F_{12}]^{2-}$, which is responsible for the strong binding of water (Fig. S29a). In contrast, such favorable interaction is not possible with the slightly positive iodine atoms of $[B_{12}I_{12}]^{2-}$. As a result, the water molecule is bound in a staggered orientation and at a longer distance from the ion, reducing the ion dipole interaction.

Optimized B3LYP/def2-tzvppd geometries of $[B_{12}F_{12}]^{2-}$+H_2O and $[B_{12}I_{12}]^{2--}$+H_2O are given in Supplemetary Tables 3 and 4. Frequency analysis indicates that the optimized geometries are global minima. The calculated binding energies are 13.50 kcal per mol (X=F) and 9.9 kcal per mol (X=I).

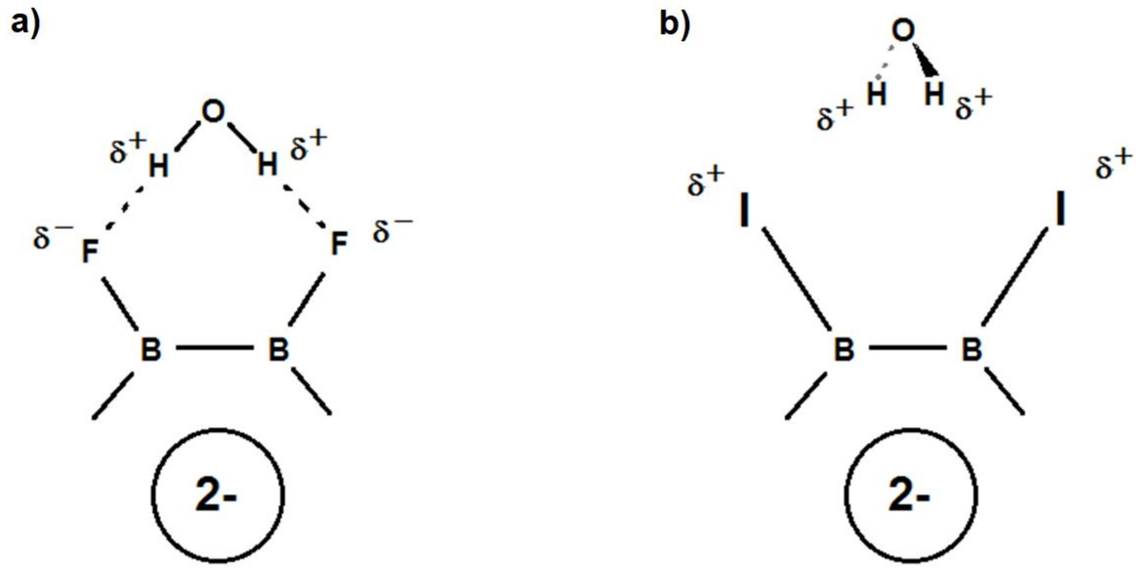

Supplementary Figure 34. Scheme illustration rationalizing the binding situation towards water in a) $[B_{12}F_{12}]^{2-}$ and b) $[B_{12}I_{12}]^{2-}$.

Supplementary Table 3: Optimized geometry $[B_{12}F_{12}]^{2-}$ +H_2O (Cartesian coordinates in Å).

B	1.45072632	0.00240044	1.17250037
B	-1.45193662	-0.00238038	-0.61830049
B	1.45193662	0.00238038	-0.61830049
B	0.89429884	1.45170915	0.27743246
B	-0.00148564	0.89586325	1.72618476
B	0.00148564	-0.89586325	1.72618476
B	0.89916184	-1.44878399	0.27738517
B	0.00150631	-0.89345238	-1.16560846

B	-0.89429884	-1.45170915	0.27743246
B	-1.45072632	-0.00240044	1.17250037
B	-0.89916184	1.44878399	0.27738517
B	-0.00150631	0.89345238	-1.16560846
F	2.62960497	0.00438284	1.90113025
F	-2.62598058	-0.00458332	-1.35301384
F	2.62598058	0.00458332	-1.35301384
F	1.61933273	2.63260439	0.27278535
F	-0.00270138	1.62470702	2.90505286
F	0.00270138	-1.62470702	2.90505286
F	1.62800939	-2.62733049	0.27298881
F	0.00254003	-1.62834891	-2.35377650
F	-1.61933273	-2.63260439	0.27278535
F	-2.62960497	-0.00438284	1.90113025
F	-1.62800939	2.62733049	0.27298881
F	-0.00254003	1.62834891	-2.35377650
H	-0.00115966	0.74661310	-4.14204502
O	0.00000000	0.00000000	-4.75618191
H	0.00115966	-0.74661310	-4.14204502

Supplementary Table 4: Optimized geometry $[B_{12}I_{12}]^{2-}$ +H_2O (Cartesian coordinates in Å).

B	1.05747708	1.40813096	0.00000000
B	-1.07099700	-1.23074534	0.00000000
B	1.64989947	-0.27216161	0.00000000
B	0.83356888	0.38517774	-1.44197427
B	-0.48601729	1.44809638	-0.89110252
B	-0.48601729	1.44809638	0.89110252
B	0.83356888	0.38517774	1.44197427
B	0.47153078	-1.26854827	0.88988714
B	-0.84736630	-0.20748407	1.44202243
B	-1.66270272	0.44963803	0.00000000
B	-0.84736630	-0.20748407	-1.44202243
B	0.47153078	-1.26854827	-0.88988714
I	2.42487788	3.10223506	0.00000000
I	-2.42729998	-2.93132523	0.00000000
I	3.77616304	-0.73988231	0.00000000
I	1.91398385	0.76251241	-3.29425403
I	-1.10159500	3.19435104	-2.03633141
I	-1.10159500	3.19435104	2.03633141
I	1.91398385	0.76251241	3.29425403
I	1.09582731	-3.01128963	2.04440581
I	-1.92597401	-0.59154867	3.29360552
I	-3.79034481	0.91038599	0.00000000

I	-1.92597401	-0.59154867	-3.29360552
I	1.09582731	-3.01128963	-2.04440581
H	0.02595746	-5.43438584	0.74781607
O	-0.01722066	-6.04343106	0.00000000
H	0.02595746	-5.43438584	-0.74781607

Supplementary Note 21: Dropcasted $[B_{12}X_{12}]^{2-}$ salt solutions.

Supplementary Figure 35 shows the crystals formed by dropping a solution of dodecaborate salt ($Cs_2[B_{12}F_{12}]$) on an FSAM surface. The exact appearance of these crystals is dependent on the counterion of the boron clusters. Independent of counterion and surface, the formed crystalline material did not change over time or under the influence of wet air.

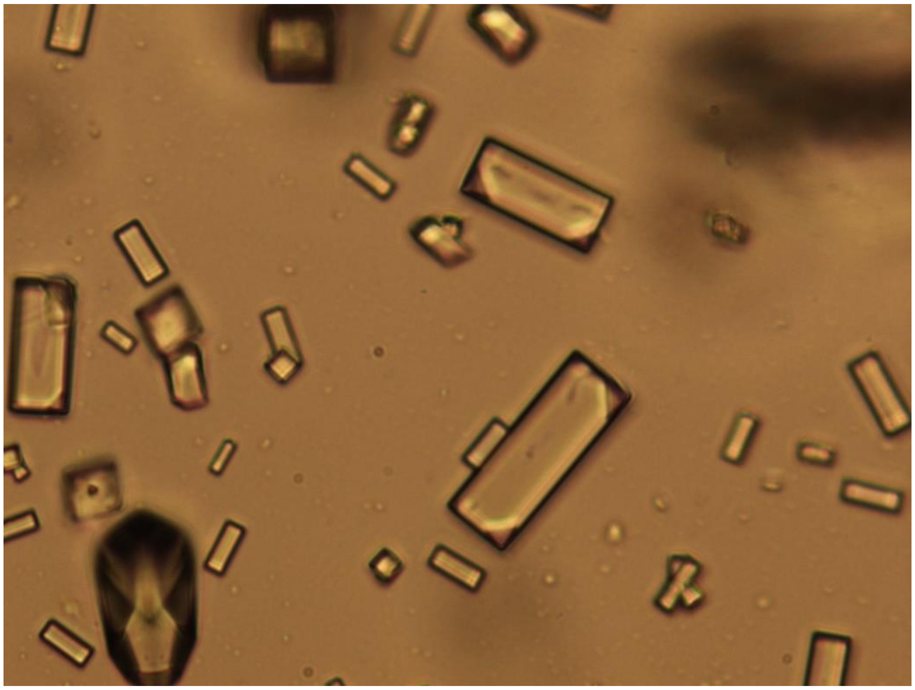

Supplementary Figure 35: Optical light microscopy image (image width 178 µm) showing crystals that have formed after dropcasting of $Cs_2[B_{12}F_{12}]$ in methanol on an FSAM surface after the solvent has evaporated.

Supplementary References:

1. Laskin, J.; Wang, P.; Hadjar, O., Soft-landing of peptide ions onto self-assembled monolayer surfaces: an overview. *Phys. Chem. Chem. Phys.* **10**, 1079-1090 (2008).
2. Johnson, G. E.; Gunaratne, D.; Laskin, J., Soft- and reactive landing of ions onto surfaces: Concepts and applications. *Mass Spectrom. Rev.* **35**, 439-479 (2016).
3. Weigend, F.; Ahlrichs, R., Balanced basis sets of split valence, triple zeta valence and quadruple zeta valence quality for H to Rn: Design and assessment of accuracy. *Phys. Chem. Chem. Phys.* **18**, 3297-3305 (2005).
4. Tiritiris, I.; Schleid, T.; Muller, K., Solid-state NMR studies on ionic closo-dodecaborates. *Appl. Magn. Reson.* **32**, 459-481 (2007).
5. Judai, K.; Abbet, S.; Worz, A. S.; Rottgen, M. A.; Heiz, U., Turn-over frequencies of catalytic reactions on nanocatalysts measured by pulsed molecular beams and quantitative mass spectrometry. *Int. J. Mass Spectrom.* **229 (1-2)**, 99-106 (2003).
6 Rappoport, D.; Furche, F., Property-optimized gaussian basis sets for molecular response calculations. *J. Chem. Phys.* **133**, 134105 (2010).
7. Grimme, S.; Ehrlich, S.; Goerigk, L.; Effect of the damping function in dispersion corrected density functional theory. *J. Comput. Chem.* **32**, 1456-1465 (2011).
8. Hu, Q. C.; Wang, P.; Gassman, P. L.; Laskin, J., In situ Studies of Soft- and Reactive Landing of Mass-Selected Ions Using Infrared Reflection Absorption Spectroscopy. *Anal. Chem.* **81**, 7302-7308 (2009).
9. Hu, Q. C.; Laskin, J., Reactive Landing of Dendrimer Ions onto Activated Self-Assembled Monolayer Surfaces. *J. Phys. Chem. C* **118**, 2602-2608 (2014).
